# Recent insights on challenges encountered with phage therapy against gastrointestinal-associated infections

**DOI:** 10.1186/s13099-025-00735-y

**Published:** 2025-08-11

**Authors:** Reem A. Youssef, Masarra M. Sakr, Rania I. Shebl, Khaled M. Aboshanab

**Affiliations:** 1 Department of Microbiology and Immunology, Faculty of Pharmacy, Ahram Canadian University, Giza, Egypt; 2 Department of Microbiology and Immunology, Faculty of Pharmacy, Ain Shams University, Cairo, 11566 Egypt

**Keywords:** Bacterial gastroenteritis, Antimicrobial resistance, Phage therapy, Challenges

## Abstract

Gastrointestinal (GI) infections, caused by pathogens such as *Escherichia coli*, *Salmonella* sp., *Shigella* sp., and *Clostridium difficile*, pose significant global health challenges due to their prevalence, severity, and increasing resistance to conventional treatments. While phage therapy offers a targeted, adaptable, and potentially safer approach for treating these infections, several challenges hinder its widespread clinical application. This review explores the current state of phage therapy for GI-associated infections, highlighting key obstacles such as phage stability in the harsh GI environment, host immune responses that can impede phage efficacy, phage-bacteria interactions, and bacterial adaptation mechanisms, such as mutations in phage receptors, which can lead to phage-insensitive mutants. Additionally, the specificity of phages to bacterial strains necessitates the development of diverse phage cocktails tailored to individual infections. The complex interactions between phages and the gut microbiome present challenges in ensuring that phage therapy does not disrupt beneficial gut bacteria. Despite these challenges, advances in phage isolation, genetic engineering, and delivery systems offer promising avenues to optimize phage therapy for GI infection. This review underscores the pharmacokinetics and pharmacodynamics of phage therapy while emphasizing the need for multidisciplinary approaches to overcome existing barriers and translate this innovative treatment into clinical practice. Accordingly, ongoing research is necessary to optimize phage delivery methods, develop effective phage combinations, and understand the interactions between phages and the gut microbiome to ensure safe and effective treatments.

## Background

Diarrheal diseases remain a major global health challenge, particularly in underdeveloped countries where contaminated water, inadequate sanitation, and malnutrition contribute to high prevalence rates. Annually, diarrheal disease affects nearly 1.7 billion children worldwide and remains the second leading cause of death among children under age five [1]. Bacterial gastroenteritis, which is a primary cause of these diarrheal diseases, can range from secretory gastroenteritis caused by pathogens like *Vibrio cholerae*, Enterotoxigenic *Escherichia coli* (ETEC), *Clostridium perfringens*, *Bacillus cereus*, and *Staphylococcus aureus*, to inflammatory gastroenteritis caused by *Shigella*, Shiga toxin-producing *E. coli* (STEC), *Salmonella* (excluding *Salmonella* Typhi/Paratyphi), *Vibrio parahaemolyticus*, *Clostridium difficile*, and *Campylobacter*. The most severe cases are caused by invasive pathogens such as *Salmonella* Typhi/Paratyphi and *Yersinia enterocolitica* [2].

Although less prevalent in high-income countries, diarrheal diseases continue to pose significant health risks, particularly due to foodborne infections. Between 2014 and 2022, the Centers for Disease Control and Prevention (CDC) in the United States (US) reported an average of 800 foodborne illness outbreaks annually, leading to approximately 15,000 cases, 800 hospitalizations, and 20 deaths each year [3]. The growing prevalence of bacterial pathogens in gastrointestinal infections is compounded by the increasing challenge of antimicrobial resistance (AMR). underscores the urgent need for novel therapies and strengthened antimicrobial stewardship to maintain effective disease control globally. Given the escalating challenge of AMR, bacteriophages have re-emerged as promising therapeutic candidates, offering a viable alternative to conventional antibiotics. These viruses are remarkably specific to their bacterial hosts [4]. Once administered, phages can multiply in the presence of susceptible bacteria, effectively auto-dosing and sustaining therapeutic activity [5]. Their encoded enzymes—like depolymerases and lysins—are adept at degrading protective biofilm matrices, enabling access to deep-seated infections [6]. Critically, when used alongside antibiotics, phages demonstrate phage–antibiotic synergy (PAS), where certain antibiotics enhance phage replication and bacterial killing, often reducing resistance development [7]. Modern advances are refining this synergy through optimized phage cocktails, dosage strategies tailored to specific pathogens, and engineered phages designed for broader host range or enhanced efficacy [8].

### Prevalence of key bacterial pathogens and the growing challenge of antimicrobial resistance

#### E. coli

Pathogenic *E. coli* is classified into extraintestinal (ExPEC) and diarrheagenic (DEC) groups. Key DEC pathotypes include Enteropathogenic (EPEC), Enterohemorrhagic (EHEC), ETEC, Enteroaggregative (EAEC), Entero-invasive (EIEC), and Diffusely Adherent (DAEC) [9]. Gastrointestinal (GIT) infections, including those caused by *E. coli* are responsible for 443,832 deaths annually from diarrheal illnesses [10], making it the second leading cause of death in this age group in underdeveloped nations, after pneumonia [11]. Healthcare infrastructure, water quality, and hygiene are considered important factors that greatly influence the prevalence of *E. coli-*associated infections [12]. EHEC infections are particularly significant in both industrialized and developing nations. STEC causes over 265,000 infections annually in the US [13], and Studies in Upper and rural Egypt found DEC in 20 and 66% of diarrhea cases in children respectively [14, 15]. The rise of antimicrobial resistance (AMR) and diverse pathogenic strains has led to widespread antibiotic resistance, with threats from carbapenemase-producing isolates (KPC-2, NDM, OXA-48-like) and extended-spectrum β-lactamases (ESBLs), often resistant to aminoglycosides, quinolones, and cephalosporins [16]. High prevalence of multidrug-resistant (MDR) isolates resistant to three or more classes has also been reported [17]. This antimicrobial resistance is multifactorial driven by genetic and adaptive mechanisms. It is primarily propagated through horizontal gene transfer of mobile genetic elements encoding β-lactamases, including ESBLs, AmpC, and carbapenemases. Enzymatic inactivation and target site alterations—such as mutations in DNA gyrase, topoisomerase IV, and ribosomal components—confer resistance to fluoroquinolones, macrolides, and β-lactams. Efflux pumps and porin mutations reduce intracellular antibiotic accumulation, while biofilm formation and stress responses enhance survival under antimicrobial pressure. Chromosomal mutations further modulate resistance to trimethoprim-sulfamethoxazole and β-lactams. Additionally, colistin resistance arises from lipid A modifications mediated by mcr genes or chromosomal regulators. These mechanisms collectively contribute to the resilience of *E. coli* against a broad range of antibiotics [18].

#### Salmonella

*S. enterica* includes six subspecies, with *enterica* being the most common, causing 99% of infections in humans and animals [19]. *Salmonella* is divided into typhoidal (TS) and non-typhoidal (NTS) types, with TS affecting humans and NTS having a broader host range, including animals [20]. NTS is a main source of foodborne infections globally, with 1.3 billion cases and 155,500 deaths annually. In 2018, it was the second leading cause of foodborne illness in the EU and the USA, and the third leading cause of death worldwide [21, 22]. In China, infections represented 70–80% of foodborne diseases [23], with symptoms ranging from gastroenteritis to more severe complications like bacteremia and osteomyelitis, particularly in vulnerable groups like children, the elderly, and immunocompromised individuals [24]. NTS is primarily zoonotic and transmitted through contaminated food, especially poultry, and also through manure-contaminated vegetables [21, 25]. Antibiotic resistance, especially due to plasmids, poses a significant public health threat [26]. Resistance to ciprofloxacin and extended-spectrum β-lactamase producers (ESBLs) have become more common, with MDR strains emerging against most of the utilized antibiotics, including ciprofloxacin, amoxicillin, ceftriaxone, ampicillin, and trimethoprim-sulfamethoxazole, which are used to treat invasive *Salmonella* infections [26–28].

TS includes *S.* Typhi, *S.* Sendai that cause typhoid fever, and *S.* Paratyphi A, B, and C that cause enteric fever, transmitted mainly through contaminated water and food, with food handlers playing a major role in its spread [29]. TS is endemic in areas with poor sanitation and affects travelers to these regions. MDR typhoidal strains appeared in the 1980s, and in the 2000s, extensively drug-resistant (XDR) typhoid emerged, resistant to cephalosporins, fluoroquinolones, and first-line antibiotics, with cases reported in Bangladesh and Pakistan [30, 31]. Since then, reports of XDR typhoid have emerged from every endemic nation, including the US [32, 33]. Despite the effectiveness of azithromycin and carbapenems, antibiotic resistance remains a challenge, with azithromycin-resistant *S. Typhi* strains also identified [34]. A recent case study of 6 years old child having Down syndrome reported pan drug resistant *S.* Typhi to all the tested antibiotics including Ampicillin, chloramphenicol, ceftriaxone, cefixime, trimethoprim-sulphamethoxazole, ciprofloxacin, meropenem, imipenem and azithromycin [35]. *Salmonella* exhibits a complex array of antimicrobial resistance mechanisms that include the production of β-lactamases and aminoglycoside-modifying enzymes, which enzymatically inactivate antibiotics. At the same time, the expression of multiple efflux pump systems (such as AcrAB–TolC and MacAB–TolC) actively expel antimicrobial agents from the cell. Additionally, mutations in target sites like DNA gyrase and topoisomerase IV contribute to fluoroquinolone resistance, while porin loss and outer membrane alterations reduce drug permeability. Biofilm formation further protects *Salmonella* populations by limiting antibiotic penetration and supporting persistent cell development. Moreover, plasmid-mediated gene transfer facilitates rapid dissemination of resistance traits across strains. Together, these mechanisms pose significant challenges to treatment and underscore the importance of novel therapeutic strategies and enhanced surveillance efforts [36].

#### Campylobacter

Campylobacteriosis has been the most commonly stated foodborne gastroenteritis in humans, with 50–80% of reported cases due to the poultry reservoir [37]. In the US. 9.4 million cases of foodborne diseases are recorded each year, whereas 9% of those infections are caused by Campylobacter species, rendering them the third most common bacterial pathogen [38]. While in the European Union (EU) campylobacteriosis is the most common foodborne bacterial illness, with 127,840 registered cases in 2021 only [39]. The two most common reported species were *Campylobacter (C.) jejuni and C. coli* (88.4% and 10.1%, respectively, in 2021) [37]. It is important to point out that finding an alternative to antibiotic therapy is crucial, especially with the recorded substantial levels of resistance to antibiotics such as ciprofloxacin and erythromycin [40]. *Campylobacter* species deploy an extensive arsenal of antimicrobial resistance mechanisms that are mediated through enzymatic inactivation—such as β-lactamases (e.g., blaOXA variants), aminoglycoside-modifying enzymes (aadE, aphA-3), and erythromycin methylases (ermB)—as well as ribosomal protection (TetO). Mutations in DNA gyrase (gyrA) and 23 S rRNA underpin fluoroquinolone and macrolide resistance, respectively. Additional defense involves the overexpression of efflux pumps (e.g., cmeABC), coupled with porin alterations that reduce drug uptake. Plasmids and other mobile genetic elements drive the dissemination of resistance determinants, while biofilm formation and adaptive stress responses bolster drug tolerance. Widespread antibiotic use in both human and animal settings has favored the propagation of these mechanisms, resulting in multidrug-resistant *Campylobacter* strains that hinder successful treatment and raise the demand for vigilant monitoring and targeted control strategies [41].

#### Shigella

*Shigella* causes shigellosis (bacillary dysentery) with *S. flexneri* being considered as one of the major causes of bacillary dysentery cases in the world especially in underdeveloped areas with poor sanitation. *Shigella* is responsible for about 5 to 15% of all diarrheal cases worldwide, including 1.1 million fatalities, especially in children under the age of five where it accounts for two-thirds of all morbidity and mortality [42].

*Shigella* isolates exhibited significant resistance to most antibiotics, with notable regional variations. In Asia, the highest resistance rates were observed for streptomycin (98.4%), trimethoprim (95.5%), and ticarcillin (90.5%), while the lowest resistance rates were for carbapenem antibiotics and nitrofurantoin, with rates close to 0% [43]. There is also a growing concern regarding the increasing resistance to third-generation fluoroquinolones, cephalosporins, and, most recently, to azithromycin, thus, this should be taken into consideration in treatment policies [44]. *Shigella* species have harnessed a sophisticated array of antimicrobial resistance mechanisms, fostering their persistent threat to public health, including the overexpression of (resistance-nodulation-cell division) RND-family efflux pumps (e.g., AcrABTolC) alongside loss or downregulation of porins, which together decrease intracellular antibiotic accumulation. They acquire target-site mutations—particularly within the quinolone-resistance–determining regions of *gyrA*, *parC*, and occasionally *parE*—conferring resistance to fluoroquinolones. Additionally, production of diverse β-lactamases, including classical and extended-spectrum types (TEM, CTX-M, SHV, OXA), degrades β-lactam antibiotics. Resistance is further exacerbated by plasmid-mediated mechanisms such as mobile elements that carry PMQR genes (*qnr*, *aac(6’)-Ib-cr*), macrolide resistance genes (e.g., *mphA*), integrons clustering multiple resistance determinants, and even colistin-resistance genes like *mcr-1*, all of which facilitate rapid horizontal gene transfer. Through the convergence of active efflux, target modification, enzymatic degradation, and plasmid-borne gene exchange, *Shigella* evolves into formidable multidrug- and extensively drug-resistant strains, underscoring the urgent need for novel treatment approaches and vigilant surveillance [45, 46].

#### Clostridium difficile

*C. difficile* produces toxins, leading to gastrointestinal conditions ranging from mild diarrhea to life-threatening complications like pseudomembranous colitis, toxic megacolon, colon perforation, and septic shock. In the US, *C. difficile* infections (CDI) affects around approximately half a million individuals each year with approximately 80,000 recurrences and 30,000 deaths [47]. In the EU/ European Economic Area, an estimated 123,997 healthcare-associated *C. difficile* infections occur annually, resulting in around 3,700 deaths [48]. Both the incidence and severity of *C. difficile* infections have increased due to the emergence of hypervirulent strains, such as the BI/NAP1/027 strain [49]. Increasing resistance to metronidazole and vancomycin has also been reported worldwide. The long-term antibiotic utilization is considered the primary risk factor for CDI, especially with antibiotics, such as clindamycin, cephalosporins, and fluoroquinolones and certain penicillins like co-amoxiclav, which can promote CDIs [48]. Resistance to other drugs like rifampin and fidaxomicin is also increasing globally [50]. *C. difficile* is responsible for approximately 15–25% of antibiotic-associated diarrhea cases, 50–75% of antibiotic-related colitis cases, and nearly all instances (90–100%) of antibiotic-associated pseudomembranous colitis [51]. In two tertiary-care hospitals in Mexico, highly drug-resistant *C. difficile* ribotypes 027 and 001 were identified, with 40.3% and 3.2% of isolates showing reduced susceptibility to vancomycin and fidaxomicin, respectively. This is particularly concerning given that fidaxomicin is not currently accessible in Mexican healthcare facilities [52]. This resistance is a multifaceted resistance that includes intrinsic and acquired adaptations. Intrinsic adaptations include biofilm production and sporulation that confer phenotypic tolerance by physically shielding cells and reducing metabolic activity. Moreover, acquired adaptations alters drug targets through mutations in RNA polymerase (*rpoB*) for rifamycins—and modify peptidoglycan precursors via mutations in the *vanG* operon homolog (*vanG_Cd*), reducing vancomycin affinity. Resistance to metronidazole arises through chromosomal changes affecting oxidoreductive and iron-dependent metabolic pathways, and, in some cases, via plasmidencoded inactivation mechanisms. Active efflux systems also extrude drugs, decreasing intracellular antibiotic concentrations. This combination of target al.teration, enzymatic drug modification, efflux, and persistence strategies has fostered the emergence of multidrug-resistant *C. difficile* strains, reinforcing the need for novel therapeutic tactics and meticulous epidemiological tracking [50].

#### Staphylococcus aureus

*S. aureus* is a significant foodborne pathogen responsible for a range of illnesses, including staphylococcal food poisoning caused by the ingestion of pre-formed enterotoxins, primarily enterotoxin A. Human carriers, particularly food handlers, are the main sources of contamination. Symptoms typically occur rapidly and include nausea, vomiting, and abdominal cramps, resolving within 24–48 h, though severe cases can affect vulnerable populations. Despite causing an estimated 241,000 cases annually in the U.S., underreporting is widespread due to the self-limiting nature of the illness and limited surveillance systems, especially in developing countries, indicating a far greater global burden than currently documented [53]. A key concern with *S. aureus* is its ability to acquire antibiotic resistance, particularly methicillin-resistant *S. aureus* (MRSA), which is driven by the mecA gene encoded on the SCCmec element. MRSA strains are typically resistant to most β-lactams, making treatment challenging and often reliant on vancomycin, although resistance to vancomycin is also emerging. MRSA has evolved beyond hospital settings to cause community-associated and livestock-associated infections, with increasing prevalence in humans and animals due to antimicrobial misuse [54]. *S. aureus* mechanisms of resistance to antimicrobials includes β-lactam resistance mediated by *mecA*-encoded PBP2a from SCC*mec* cassettes, alongside β-lactamases and occasional PBP mutations; glycopeptide resistance, featuring *vanA* acquisition or cell wall thickening in VISA strains that reduce vancomycin accessibility; oxazolidinone resistance, driven by Cfr- and Erm‐mediated 23 S rRNA methylation and ribosomal mutations; aminoglycoside resistance, through plasmid‐encoded modifying enzymes (acetyl-, phospho-, nucleotidyltransferases); fluoroquinolone, tetracycline and MLS‐B resistance via gyrA/gyrB/parC/parE mutations and overactive efflux pumps (NorA, MdeA, MepA from MATE (multidrug and toxic compound extrusion), MFS (major facilitator superfamily), SMR (small multidrug resistance) and ABC (ATP-binding cassette) families); and other mechanisms including resistance to rifampicin, chloramphenicol, fusidic acid, mupirocin, and daptomycin. This formidable genetic toolkit is compounded by active efflux systems that also support biofilm formation, vancomycin sequestration, and integration of resistance genes on mobile genetic elements—SCC*mec*, plasmids, integrons, transposons—facilitating horizontal transfer. Cumulatively, these mechanisms have given rise to multidrug- and extensively drug-resistant *S. aureus*, underscoring the imperative for novel therapeutic options and rigorous epidemiological surveillance.

#### Vibrio cholerae (VC)

Among VC strains, those belonging to serogroups O1 and O139 are linked to cholera outbreaks due to the production of cholera toxin and toxin-coregulated pilus, which are crucial for intestinal colonization and diarrhea [55]. Cholera remains endemic in regions like Asia, Africa, Central and South America, and Bangladesh, with outbreaks primarily linked to the El Tor (O1) biotype [56]. AMR in VC emerged in the 1970s, with resistance to polymyxin B and other antibiotics observed, particularly in the El Tor biotype. Resistance patterns vary globally, with selective antibiotic pressures influencing the spread of resistance genes [57]. In sub-Saharan Africa, VC isolates mostly belong to the O1 El Tor biotype, with common resistance to trimethoprim-sulfamethoxazole, ampicillin, chloramphenicol, and streptomycin [58, 59]. VC exhibits a wide array of AMR mechanisms that contribute to its persistence and treatment failure in clinical settings. Key strategies include active efflux of antibiotics via multiple transporter families—such as MATE, MFS, RND, SMR and ABC—many of which also support virulence and environmental adaptation. Efflux pumps like NorM, VcmA, and EmrD-3 are implicated in resistance to a broad range of drugs. Resistance is also mediated by chromosomal mutations, particularly in the quinolone resistance-determining regions (QRDRs) of *gyrA* and *parC*, leading to reduced susceptibility to fluoroquinolones. Additionally, VC acquires β-lactamase genes, including *blaNDM-1*, which confer resistance to β-lactams and carbapenems. Structural modifications, such as lipid A alteration by the *almEFG* operon, contribute to polymyxin resistance. Outer membrane impermeability further limits antibiotic entry, particularly for large molecules, while inducible stress responses and enzymatic degradation of drugs add to the problem of resistance. These combined mechanisms underscore the adaptive plasticity of VC under antimicrobial pressure and highlight the urgency of continuous surveillance and novel therapeutic strategies [57].

#### Yersinia enterocolitica (Y. enterocolitica)

*Y. enterocolitica* primarily causes yersiniosis, leading to gastrointestinal illness, mesenteric lymphadenitis, and endocarditis, particularly in children [60]. Infections typically result from consuming contaminated food, especially raw or undercooked pork, as pigs are the primary reservoir [61]. The bacterium is biochemically and serologically diverse, with serotype O8 is the most virulent and can sometimes cause severe gastrointestinal ulceration or even death [60]. Yersiniosis is the third most reported zoonotic infection in the EU, though actual cases may be underreported [62]. *Y. entericolitica* develops antimicrobial resistance through multiple mechanisms, primarily via β-lactamase production (BlaA and BlaB), which hydrolyze β-lactam antibiotics [63]. Efflux pumps, notably the AcrAB-TolC system, actively expel diverse antibiotics such as tetracycline resistance typically results from plasmid-borne tet genes (e.g., tet(A), tet(B)) that encode efflux pumps and ribosomal protection proteins [63, 64]. Target site modifications, including mutations in *gyrA* and *parC*, reduce fluoroquinolone efficacy, while changes in membrane porins limit drug entry [63]. Furthermore, horizontal gene transfer via plasmids, integrons, and transposons (e.g., Tn2670) facilitates the acquisition and dissemination of resistance genes, enhancing the survival under antimicrobial pressure and increasing the public health risks. Aminoglycoside resistance is mediated by enzymatic inactivation via acetyltransferases, phosphotransferases, or nucleotidyltransferases [64]. Together, these mechanisms—enzymatic drug inactivation, decreased permeability, target modification, and gene mobility—underpin the adaptive resistance landscape of this pathogen.

#### Helicobacter pylori (H. pylori)

Gram-negative *H. pylori* is a major cause of gastrointestinal infections, affecting around 50% of the global population [65]. Infections with *H. pylori* range from peptic ulcers and gastritis to more serious problems such as gastric adenocarcinoma and mucosa-associated lymphoid tissue lymphoma. In addition, *H. pylori* infection has been connected to biliary tract malignancies, which is quite intriguing [66]. Even though *H. pylori* only colonizes the stomach epithelium, the infection has also been linked to several extra-gastric illnesses, such as colorectal cancer (CRC). Direct causative and functional linkages between the chronic infection and CRC have only recently been discovered, despite epidemiological studies reporting a nearly two-fold greater risk for infected persons to acquire CRC [65]. *H. pylori* exhibits antimicrobial resistance primarily through chromosomal mutations and adaptive mechanisms that impair antibiotic efficacy. Resistance to clarithromycin is most commonly associated with point mutations in the 23 S rRNA gene (A2142G, A2142C, A2143G), which prevent effective ribosomal binding [67]. Fluoroquinolone resistance results from mutations in the *gyrA* and *gyrB* genes within the quinolone resistance-determining region (QRDR), while metronidazole resistance involves loss-of-function mutations in *rdxA* and *frxA*, which encode enzymes essential for drug activation [68]. Tetracycline resistance is less common but arises through alterations in the 16 S rRNA gene that interfere with ribosomal interaction [68]. Additionally, reduced membrane permeability and overexpression of efflux pumps contribute to decreased intracellular antibiotic concentrations, further enhancing resistance and contributing to treatment failure which poses a significant threat to public health [68].

### Less-studied pathogens associated with gastroenteritis

Among the well- known pathogens that cause gastroenteritis, there are other less studied pathogens that play a key role in this type of infection. For example, *Aeromonas* spp is primarily *Aeromonas* spp, is primarily associated with aquatic environments and fish disease. These bacteria are widely distributed in freshwater, soil, and food, thus enabling frequent human contact. In the meantime, clinically relevant species such as *A. caviae*, *A. hydrophila*, and *(A) veronii* have been isolated from fecal, blood, bile, and wound samples, with infections ranging from mild gastroenteritis to severe conditions like sepsis and peritonitis. Despite being underreported, *Aeromonas* infections can mimic other gastrointestinal diseases and may affect both immunocompromised and healthy individuals. Diagnostic challenges arise due to limitations in conventional biochemical identification, necessitating molecular methods. *Aeromonas* strains harbor various virulence factors and often show resistance to β-lactam antibiotics, highlighting their role as reservoirs of antimicrobial resistance [69]. Moreover, *Plesiomonas shigelloide*s is a Gram-negative, rod-shaped bacterium recognized for its involvement in gastrointestinal disorders, including acute secretory gastroenteritis and a clinical syndrome resembling shigellosis. Beyond intestinal disease, this organism has been implicated in various extraintestinal infections, such as bacteremia, meningitis, and conditions mimicking appendicitis. Its primary ecological niche includes freshwater and estuarine systems, and it has been isolated from a wide range of environmental and animal sources, including surface water, wild fauna, and domesticated species [70]. Few studies reported the antimicrobial resistance among *Plesiomonas shigelloides* strains isolated from clinical samples and environmental sources. A recent study reported resistance to amikacin, trimethoprim-sulfamethoxazole, chloramphenicol, tetracycline, cefazolin, streptomycin, and florfenicol [71]. Furthermore, *Edwardsiella tarda.* is a less studied pathogen causing human infections. Human infections caused by *E. tarda* are most prevalent in subtropical regions and present mainly as gastrointestinal illness, septicemia, and wound infections [72]. Although earlier studies indicated that *E. tarda* is typically susceptible to many antibiotics, it is intrinsically resistant to tetracyclines, aminoglycosides, quinolones, fosfomycin, most β-lactams (including penicillin), colistin, and polymyxin (B) However, emerging clinical reports have documented resistant strains to antimicrobial agents such as ampicillin, quinolones, gentamicin, piperacillin-tazobactam, and carbapenems—possibly due to widespread antibiotic use in aquaculture [73].

### Bacteriophages and their capabilities

**Fig. 1:**
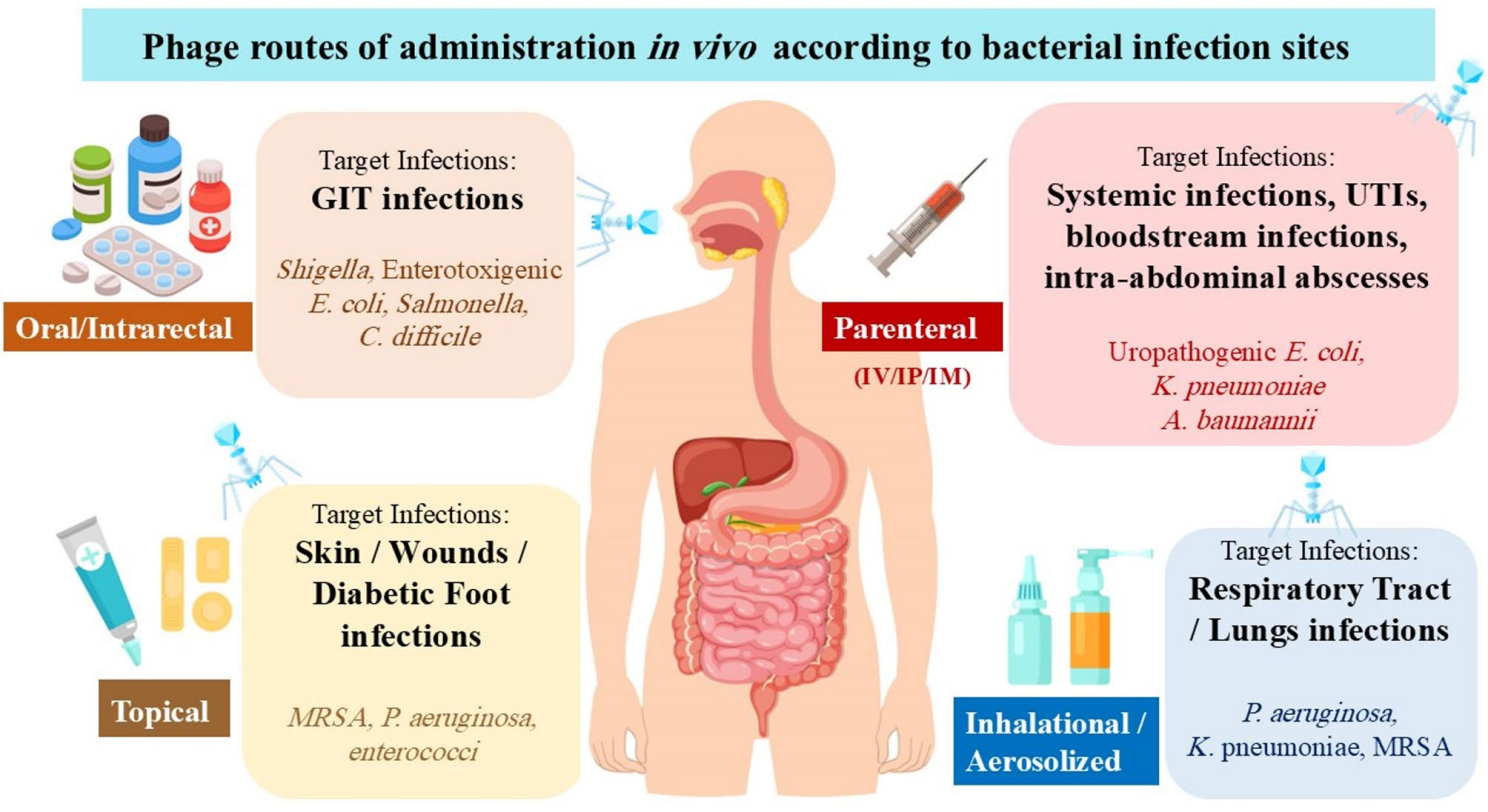
Routes of administration of bacteriophages in vivo according to bacterial infection sites

On our globe, bacteriophages are among the most abundant and ubiquitous biological entities, found in virtually every ecosystem, particularly those inhabited by bacteria [74]. Bacteriophages are viruses that exclusively infect and replicate within their bacterial host which makes them attractive biological tools for bacterial control [75]. With their high bacterial host specificity, self-replication, self-limitation, constant adaptation, low intrinsic toxicity, and ease of isolation from the environment, phage therapy has resurfaced as a viable option in the era of growing antibiotic resistance [76]. Phage therapy (PT) is the term used to describe the clinical administration of lytic phages directly to a patient (human or animal) to lyse the bacterial pathogens [77]. It relies on the basic characteristics of lytic phages, which selectively infect living bacteria, replicate to the greatest extent possible, and then propagate their progeny by rupture of the bacterial membrane, which ultimately results in the death of the host bacterial cell [78]. Bacterial membrane surface receptors are necessary for phage infection and provide the benefit of restricting phage infectivity to specific bacterial hosts. PT provides several notable benefits in comparison to conventional antibiotics, particularly in terms of minimizing adverse drug interactions within living systems and reducing the risk of antibiotic-associated toxicity [79]. A major advantage lies in its ability to target and disrupt bacterial biofilms—a protective build-up of extracellular polymeric substances (EPS) secreted by a colonization of bacteria—which confer protection against both human host defenses and antibiotic treatment [80]. Bacteriophages are naturally equipped, or can be genetically modified, to express biofilm-degrading enzymes such as polysaccharide depolymerases that facilitate the breakdown of EPS, enhancing phage diffusion and enabling the lysis of embedded bacterial cells [78]. Once inside the biofilm, phages replicate and propagate through successive bacterial infections, contributing to a cascading antimicrobial effect [78]. Moreover, PT can be customized according to the type of pathogen and the site of infection to maximize therapeutic efficacy for the patient. Phages can be administered via various routes, including oral, topical, parenteral, and respiratory methods, such as inhalation or nebulization [81], as shown in Fig. 1.

### Oral phage therapy (OPT)

Oral phage therapy (OPT) is gaining attention as a valuable strategy for managing gastrointestinal (GI) infections, largely due to its precision in targeting harmful bacteria while leaving beneficial gut flora intact [82]. OPT not only eliminate bacterial pathogens such as *E. coli* from the intestinal tract, but also plays a role in extraintestinal infections, including urinary tract infections (UTIs) [83]. Compared to injectable routes, oral delivery is less invasive and easier to administer, which improves patient compliance. It also allows phages to multiply directly at the infection site, potentially increasing treatment effectiveness with fewer doses [78]. In terms of safety, OPT has been shown to provoke minimal immune responses and is generally well tolerated in clinical settings, with only mild, transient side effects reported [84]. Advances in encapsulation technology have further enhanced the stability of phages during their passage through the acidic gastric environment, reinforcing OPT’s potential as a scalable, low-cost option in combating antibiotic-resistant infections [78]. For example, two clinical cases of chronic bacterial prostatitis caused by MDR bacteria demonstrated the effectiveness of OPT after repeated antibiotic failure. In both cases, tailored bacteriophage treatments—administered orally and rectally—targeted resistant strains like *E. coli* and *Enterococcus faecalis*. Treatment was guided by phage sensitivity testing and delivered in structured cycles. Both patients achieved significant symptom relief and bacterial eradication. These outcomes emphasize the potential of OPT as a practical and targeted strategy for treating antibiotic-resistant infections, particularly in hard-to-reach sites like the prostate [85]. Moreover, another clinical trial involved a healthcare worker developed a urinary tract infection whose gastrointestinal tract was colonized with MRSA, leading to a secondary UTI by the same strain. After receiving oral phage therapy specifically targeting MRSA, both the intestinal colonization and the UTI were completely resolved [86]. These case studies highlight the practical effectiveness of oral bacteriophage treatment in eliminating gastrointestinal and extraintestinal infections.

### In vitro studies for phage therapy

According to numerous studies, PT proved effective in treating a variety of MDR bacteria that cause GIT infections in vitro. A research study indicated that Phage A221, belonging to the Ackermannviridae family, specifically the Aglimvirinae subfamily and *Agtrevirus* genus, possesses a genome of 153,297 base pairs. This phage demonstrated the potential to effectively suppress the growth of *E. coli* strain GXXW-1103 in vitro for 16 h [87]. Another study revealed a synergistic antimicrobial effect between bacteriophage T4 and the antibiotic cefotaxime, and demonstrated significant in vitro clearance of biofilms formed by the T4 host strain, *E. coli* 11,303 [88]. In another research, six phages active against *E. coli*, were examined individually as well as in combinations, focusing on their host range, stability, reproduction, and in vitro effectiveness. The six-phage cocktail successfully inhibited biofilm formation in certain *E. coli* strains, although it was not effective against all strains. Interestingly, phage-resistant variants emerged when bacterial cells were exposed to a single phage, whereas this resistance did not occur in cells challenged by a combination of four or six phages [89]. A study showed that a cocktail of two phages, ELY-1 (targeting *E. coli*) and phSE-5 (targeting *S.* Typhimurium), significantly reduced bacterial counts: ELY-1 achieved a maximum reduction of 4.5 log colony-forming unit (CFU)/mL against *E. coli*, while phSE-5 reduced *S.* Typhimurium by 2.6 log CFU/mL. When tested together, the phage cocktail was more effective against *S.* Typhimurium and the mixed bacterial population, achieving a maximum reduction of 3.2 log CFU/mL, while maintaining similar effectiveness against *E. coli* as ELY-1 alone [90].

Another study reported that phages (ɸ) SaI_NFG_5581 and SaI_NFG_5577 were identified as purely lytic against *S.* Infantis, exhibiting distinct bacteriolytic activity and genetic characteristics. Both phages belong to the class of Caudoviricetes, but with different families and genome length. They were regarded as harmless because genomic study ruled out the presence of genes related to lysogeny, toxicity, or antibiotic resistance. Phages can withstand a wide range of temperatures (4 °C to 50 °C) and pH (4–10). After two hours of treatment, both ~ SaI_NFG_5581 and ~ SaI_NFG_5577 were able to reduce *Salmonella* counts in vitro by approximately 2.2 and 3.4 log CFU/mL, respectively, at 0.1 multiplicities of infection (MOI) [91]. In another study, Shang and colleagues isolated five *Salmonella* phages from soil samples, with vB_SalP_TR2 was recorded for its potential lytic activity against *Salmonella* serovar Albany and other strains, including Corvallis, Newport, Kottbus, and Istanbul. This phage is classified within the Podoviridae family based on morphological analysis and showed a latent period of 15 min and a burst size of 211 plaque forming unit (PFU) per cell. Database searches revealed 35 gene products from 96 predicted open reading frames, with no virulence or antibiotic resistance properties. vB_SalP_TR2 showed activity at different pH and temperature ranges, thus regarding it as a potential biological control agent. It effectively lysed *S.* Albany at a variety of infection multiplicities (ranging from 0.0001 to 100) after 24 h at 37 °C. Additionally, vB_SalP_TR2 significantly reduced the presence of *S.* Albany in food samples, such as milk and chicken meat, compared to control groups. These findings suggest that vB_SalP_TR2 could serve as an effective antibacterial agent for controlling *Salmonella* in food [92]. In another study, Pelyuntha and his team used a cocktail of three phages—WPX5, WPX8, and WPX9—to reduce *Salmonella* counts by three log units in vitro when applied post 6 h at MOI of 10^4 and 10^5. The phage cocktail also reduced *Salmonella* attachment on food contact surfaces, so it could be used as an effective biocontrol agent against toxin-producing, biofilm-forming, and MDR *Salmonella* in the poultry industry [93].

A study reported that group II phage vB_CcM-LmqsCPL1/1 and group III phage NCTC 12,673 when combined, the in vitro growth reduction against the *C. jejuni* target strain BfR-CA-14,430 was considerably greater than when the phages from a single group were applied alone or in combinations [37]. Another study focused on examining the effects of newly isolated group II and group III phages, both individually and in combination, on *Campylobacter* field strains. It was found that a mixture of group II phage vB_CcM-LmqsCP218-2c2 and group III phage vB_CjM-LmqsCP1-1 holds the most potential for practical use against *C. coli* and *C. jejuni* [94]. A recent study stated that a novel *C. jejuni* phage, named vB_CjeM_WX1 (WX1), was isolated from chicken feces, and it was capable of lysing 35 strains of *C. jejuni*, all of which were highly virulent and MDR. Among these, 10 strains exhibited strong biofilm formation, a key factor in bacterial survival and resistance to environmental stressors. The phage achieved a lysis rate of up to 47.3% across 76 *C. jejuni* strains. Phage WX1 inhibited the growth of the MDR and the highly virulent strain (*C. jejuni* 178-2B). It also significantly reduced biofilm formation on stainless steel, polyethylene, and glass surfaces [95]. In another study, the *Campylobacter*-specific lytic phage CP6 was isolated from chicken feces. It showed a broad host range, infecting 97.4% of different MDR *Campylobacter* isolates. In vitro, phage CP6 exhibited strong antimicrobial effects on MDR *Campylobacter*, reducing the CFUs of the bacterial host by up to 1 log compared to the control in artificially contaminated chicken breast meat. These findings suggest that phage CP6 holds potential as an effective antimicrobial agent for addressing MDR *Campylobacter* in food processing [96]. In another study, two lytic bacteriophages, namely vB_CjP and vB_CcM, were isolated and assessed for their ability to target MDR *C. jejuni* and *C. coli*, respectively. Host range testing showed that vB_CjP could infect 5 out of 10 *C. jejuni* isolates, while vB_CcM could infect 4 out of 10 *C. coli* isolates. Both phages demonstrated effective lytic activity against planktonic *Campylobacter in vitro*, indicating their potential for controlling *Campylobacter* growth. Additionally, the lytic activity of vB_CjP and vB_CcM was evaluated at variable MOIs against MDR *Campylobacter* strains. Both phages slightly delayed bacterial growth, with the highest efficiency observed at an MOI of 1. These results highlight the potential of these phages for therapeutic applications to significantly reduce *Campylobacter* spp. counts [97].

In another study, a novel bacteriophage, Sfk20, was isolated from water sources in a diarrheal outbreak area in Kolkata, India, and showed lytic activity against various *Shigella* species including *Shigella flexneri*, *Shigella sonnei*, and *Shigella dysenteriae*. Phage-host interaction and lytic activity were confirmed using ultrathin sectioning and TEM. Genomic analysis was done, and classified it as a T4 myoviridae phage. Sfk20 also demonstrated anti-biofilm activity against *Shigella* species, suggesting its potential as a biocontrol agent [98]. A research study characterized KFS-EC3, a polyvalent and lytic bacteriophage isolated from slaughterhouse sewage, capable of efficiently infecting *E. coli* O157:H7, *Salmonella* spp., and *Shigella sonnei*. It inhibited *E. coli* O157:H7 growth by 2 logs over 52 h at a low MOI of 0.001 [99]. Genomic analysis revealed a 167,440 bp genome with 273 functional genes, with no associations to antibiotic resistance, virulence, allergenicity, or lysogenicity. The isolated phage (KFS-EC3) was classified under the *Tequatrovirus* genus of the Myoviridae family, and showed promise for simultaneous pathogen control in food applications over extended periods [99]. A recent study isolated two novel phages from sewage, S2_01 and S2_02, both displaying lytic activity against *Shigella* species. A combination of these phages (1:9 ratio) showed the most effective and sustained lysis for up to 24 h. The phage cocktail inhibited and disrupted biofilms on surfaces used in food processing, including polystyrene microplates and stainless steel, by 79.29% and 42.55%, respectively, and significantly reduced the growth of planktonic cells. It was also effective against various foodborne pathogens, including six *Shigella* species, and showed potential as a biological control agent for improving food safety [100].

A study isolated four new *C. difficile* myoviruses, with ΦCD1801 showing the broadest host range, infecting 93.8% of *C. difficile* RT 078 strains. To create an effective phage therapy for CDI, a cocktail of phages targeting all *C. difficile* ribotypes is needed [101]. In another study, host range analysis of six myoviruses and one siphovirus was conducted on 80 *C. difficile* strains from 21 major epidemic ribotypes. The phages demonstrated complementary coverage, effectively lysing 18 ribotypes and 62 strains. Single-phage treatments reduced bacterial load in ribotype 076, 014/020, and 027 initially, but phage-resistant colonies emerged, which were still susceptible to infection by unrelated phages. However, combinations of specific phages completely lysed *C. difficile* and prevented the emergence of resistant or lysogenic clones [102]. A research team used a batch fermentation system to assess the effects of FCD27 phage therapy on *C. difficile* levels and related toxins, with two treatment schedules; remedial and prophylactic. The remedial treatment reduced *C. difficile* CFUs significantly, but the difference was minor, and no notable effect was seen at 48 h. Prophylactic treatment led to a 1.77 log 10 decrease in *C. difficile* at 48 h with a MOI of 7. When the MOI was increased to 10 in follow-up experiments, no viable C. difficile was detected at 48 h [103]. A research group investigated the use of the bacteriophage FCD27 in a human colon model with *C. difficile* infection. Their findings revealed a significant reduction in *C. difficile* cells and toxin production after phage treatment, without harming commensal bacteria. The study highlights the potential of phage therapy while pointing out the challenges associated with phages that have lysogenic properties [104]. A study isolated and characterized a lytic *Y. enterocolitica*-specific phage, KFS-YE from poultry, as a potential biocontrol agent. It showed potential specificity for *Y. enterocolitica* and stable lytic activity across a broad range of pH and temperatures for 40 weeks under various storage conditions. In vitro, the phage was effective against *Y. enterocolitica* at low infection doses, thus demonstrating its potential as a biocontrol agent in food applications [105].

Moreover, Abdel-Haliem and Askora isolated two lytic phages active against for *H. pylori* from wastewater [106]. Another study identified a new *H. pylori* phage, KHP30, that was stable in the acidic stomach environment, infecting 63.6% of clinical *H. pylori* isolates. The phage had a latent period of 140 min, shorter than the bacterium’s doubling time, and a small burst size of around 13. Despite having an integrase gene, KHP30 persisted as an episome in *H. pylori* strain NY43. Protein analysis identified seven potential virion proteins, and its genomic structure differs from known spherical phage families, suggesting KHP30 as a new, unclassified phage [107]. A recent study by Cuomo et al. investigated a lytic phage for *H. pylori* isolated from gastric biopsies. This phage, when combined with lactoferrin and hydroxyapatite nanoparticles, showed enhanced activity and stability in acidic environments, making it a promising therapeutic option. However, the minimum effective doses of this combination were not determined [108].

### In vivo pre-clinical studies using oral phage therapy (OPT)

Numerous studies confirmed the efficiency of OPT in vivo. A research group isolated phage A221 that was shown to effectively treat *E. coli* GXXW-1103 infections in weaned piglets. Treatment with oral microencapsulated phage A221 led to improved body weight, reduced bacterial load, and healed intestinal lesions. The phage’s therapeutic effects were like those of the antibiotic Florfenicol. These results suggest that phage A221 is a promising treatment for bacterial diarrhea in piglets and support the potential use of phage therapy in clinical settings [87]. A study developed a novel three-phage cocktail (SPFM17, ST-W77, and SE-W109) to combat bacteriophage resistance and tested it in a *Galleria mellonella* larva model infected with *Salmonella*. The phage treatment successfully eradicated *Salmonella* within 24 h and maintained this effect for 72 h. When phages were administered two hours before bacterial exposure as prophylactic treatment, all larvae survived. Phage treatment given alongside or after bacterial exposure also increased survival rates, though it was less effective than the prophylactic regimen [109]. A recent study isolated and evaluated two lytic bacteriophages, VB_ST_E15 and VB_ST_SPNIS2, that were active against MDR *Salmonella* serovars, including *S.* Typhimurium, *S.* Paratyphi A, and *S.* Typhi. In a murine model, the phage cocktail, either in its free form or microencapsulated form, reduced bacterial load, improved weight gain, and showed positive histopathological effects. These results suggest that the encapsulated phages represent a promising option for treating MDR *Salmonella* infections, warranting further clinical investigation [110].

Another research team isolated a new phage, vB_ShiP-A7, using MDR *Shigella flexneri* as the host. It belongs to the Podoviridae family, it has a latency period of 35 min, and a burst size of 100 phage particles per infected cell. The phage’s genome was 40,058 base pairs long, with no genes related to lysogeny, virulence, or antibiotic resistance. vB_ShiP-A7 effectively inhibited the growth of MDR clinical strains of *S. flexneri* and *E. coli* in biofilms. In a mouse infection model, it reduced *S. flexneri* by 3 to 10 times. This stable phage showed promising potential for treating infections caused by MDR *S. flexneri* and *E. coli* [111]. A research study used phage VMJ710, which was isolated during a 2015 cholera outbreak in Chandigarh, to effectively target MDR *V. cholerae* O1 strains. It reduced bacterial density significantly within 4–6 h and showed no resistance over 20 h of testing. The phage is genetically similar to the ICP1 phage and remains stable under varying environmental conditions. It also disrupts bacterial biofilms and reduces bacterial growth and dispersal. In an in vivo mouse model, VMJ710 significantly reduced bacterial counts after inoculation with *V. cholerae* O1. With no virulence or antibiotic resistance genes, VMJ710 shows promise as an oral treatment for cholera [112].

Phage X1, a lytic phage, was isolated and found to effectively target 27 out of 51 *Y. enterocolitica* strains. It showed high stability across a wide range of temperatures and pH, and was resistant to acidic conditions and enzymatic degradation, making it suitable for oral administration. In animal studies, a single oral dose of phage X1 significantly reduced bacterial levels in mice and improved intestinal health, with a notable reduction in proinflammatory cytokines. These results suggest phage X1 as a promising treatment for *Y. enterocolitica* infections [113]. The previously demonstrated in vivo pre-clinical studies were summarized in Table 1.

**Table 1:** Pre-clinical studies demonstrating the application of oral phage therapy against different bacterial pathogens

Animal model	Microorganism	Phage	Results	Reference
Weaned piglets	*E. coli*	Microencapsulated phage A221	Phage A221 notably enhanced the daily weight gain in piglets, decreased bacterial levels in tissues, and alleviated intestinal damage, delivering therapeutic results comparable to the antibiotic florfenicol.	[87]
Galleria mellonella larva model	*Salmonella*	A novel three-phage cocktail (SPFM17, ST-W77, and SE-W109).	Isolated phage was more effective in prophylactic regimen than treatment regimen.	[109]
Murine model	MDR *Salmonella* serovars, including *S.* Typhimurium, *S.* Paratyphi A, *and S.* Typhi	Two-phage cocktail, VB_ST_E15 and VB_ST_SPNIS2 (microencapsulated and free form).	Phage cocktail either in its free form or microencapsulated form reduced bacterial load, improved weight gain, and showed positive histopathological effects.	[110]
Mouse infection model	MDR clinical strains of *S. flexneri* and *E. coli*, including in biofilms	A new phage, vB_ShiP-A7	vB_ShiP-A7 effectively inhibited the growth of MDR clinical strains of *S. flexneri* and *E. coli*, including in biofilms.	[111]
Mice infection model	*V. cholerae* O1	Phage VMJ710	VMJ710 significantly reduced bacterial counts after inoculation with *V. cholerae* O1.	[112]
Mice infection model	*Y. enterocolitica*	Phage X1	A single oral dose of phage X1 significantly reduced bacterial levels in mice and improved intestinal health, with a notable reduction in proinflammatory cytokines.	[113]

### Clinical trials using oral phage therapy (OPT)

In 1963, the Eliava Institute conducted a Phase III clinical trial in Georgia involving 30,769 children to evaluate the activity of oral *Shigella* phages to prevent diarrhea. The double-blind, placebo-controlled study showed that oral *Shigella* phages effectively prevented diarrhea in children, but the results were published briefly with limited data. Unlike *E. coli*, *Shigella* is a simpler pathogen for phages to target, and it replicates in the colon, thus enhancing phage efficacy. Moreover, the Eliava trial focused on prevention, while subsequent studies were treatment-focused, where phages may be less effective once the disease has progressed [114]. Another study tested oral *E. coli* phage T4 on 15 healthy adults using low and high doses and found that phage presence in feces was dose dependent. The high-dose group had fecal phage one day after exposure, while only 50% of the low-dose group showed it. No fecal phage was detected a week after treatment, and phage did not impact fecal *E. coli* counts or replicate on commensal bacteria. There were no adverse effects, and no T4 phage or antibodies were found in the serum. This study is the first to assess the safety of oral phage therapy in humans [84].

Moreover, Nestlé Research conducted a phase I/II clinical trial in Bangladesh to test an oral T4-like coliphage cocktail for *E. coli*-related childhood diarrhea. The trial, which was placebo-controlled and double-blind, compared Nestlé’s phages with a product from the Russian company Microgen. While no adverse effects were observed, the trial was halted after an interim analysis showed no significant improvement in diarrhea severity. Possible reasons for the lack of efficacy include reduced OPT effectiveness without antacids, difficulty in phage replication in the intestine, low pathogen load, and the complexity of *E. coli* infections. Despite this, the researchers believe phage therapy still holds potential and further research is needed [115]. Furthermore, a recent research study evaluated the safety and tolerability of ShigActive™, a lytic bacteriophage preparation targeting *Shigella* spp., in a randomized, placebo-controlled, double-blind first-in-human Phase 1 clinical trial. Ten participants were randomly assigned to receive either ShigActive™ or a placebo, co-administered with sodium bicarbonate, orally three times daily for 7 days. Adverse events (AEs) were monitored for 29 days. 50% of participants receiving ShigActive™ reported mild gastrointestinal (GI) symptoms, and one experienced moderate fatigue. No serious or medically attended AEs were reported by day 90. There were no significant differences in GI-associated inflammatory markers or fecal microbiome changes between the ShigActive™ and placebo groups or from baseline values [116]. A summary regarding the clinical trials performed using oral phage therapy was briefly listed in Table 2.

**Table 2:** Clinical trials involving the application of oral phage therapy against different bacterial pathogens

Trial type	Microorganism	Phage	Results	Reference
Phase III clinical trial (Eliava Institute, 1963)	*Shigella*	Oral *Shigella* phages	A significant reduction in dysentery and diarrhea, particularly in younger children	[114]
Safety trial	*E. coli*	Oral *E. coli* phage T4	Oral *E. coli* phage therapy was found safe in humans, suggesting its potential for treating diarrheal diseases	[84]
Phase I/II clinical trial	*E. coli*	Oral T4-like coliphage cocktail	No significant improvement in diarrhea severity	[115]
Phase 1 clinical trial (safety trial)	*Shigella*	ShigActive™	ShigActive™ is safe and well-tolerated when taken orally, with no major differences compared to the placebo	[116]

### Challenges facing the application of OPT in the treatment of bacterial gastroenteritis

**Fig. 2:**
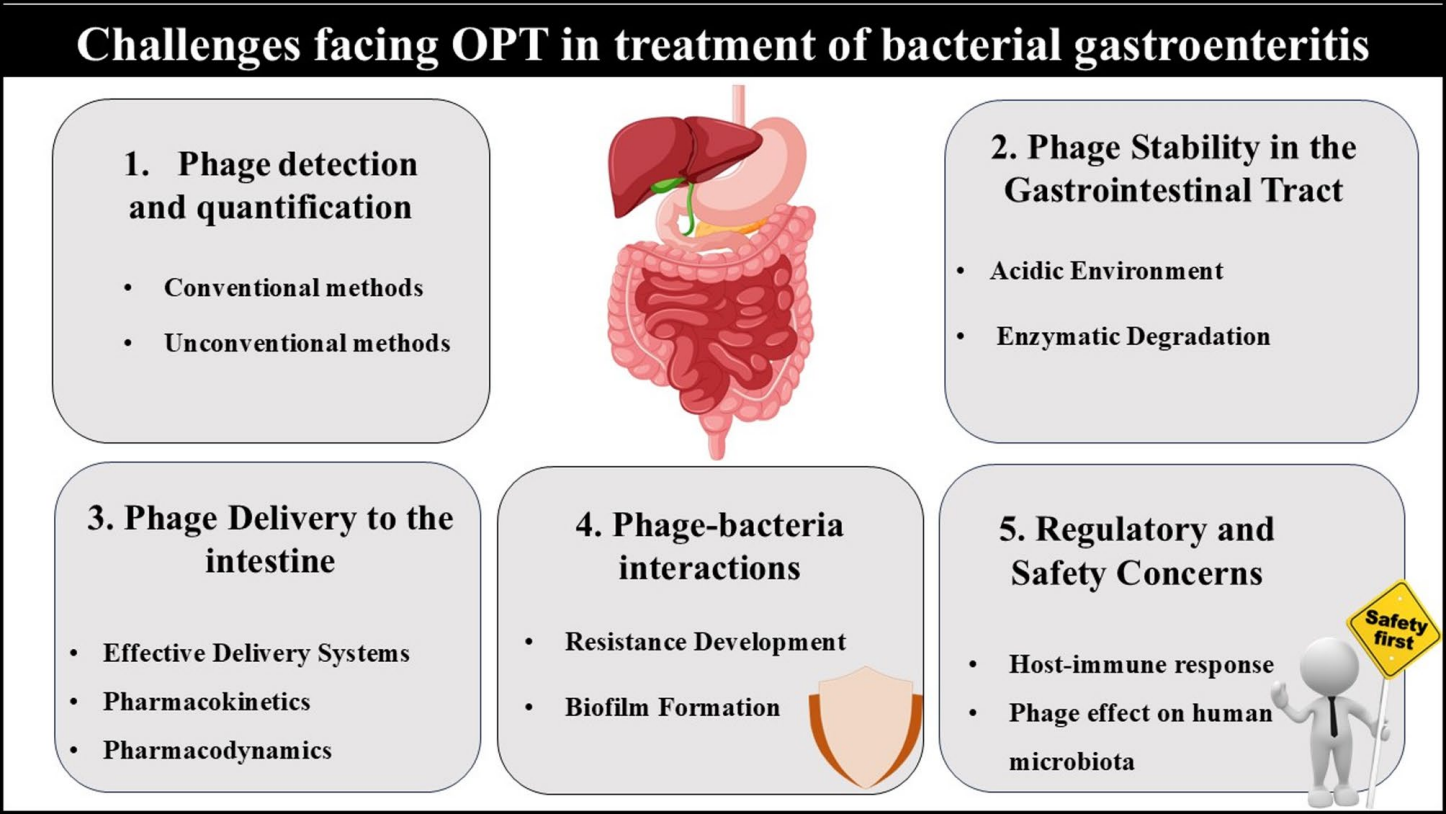
Challenges facing OPT in the treatment of bacterial gastroenteritis

Despite their capabilities, there are major challenges to overcome to put phage therapy into reality. Challenges facing the application of OPT in the treatment of bacterial gastroenteritis are shown in Fig. 2.

### Phage quantification and detection challenge

Phage detection and quantification methods vary in approach and application. The Double Agar Overlay (DLA) method is the gold standard, detecting infective phages by observing plaque formation on bacterial lawns, though it requires optimization and can be influenced by errors. Alternatives like Transmission Electron Microscopy (TEM), Flow Cytometry, and NanoSight offer rapid detection of whole phage particles but may be costly or require high sample concentrations. Molecular methods such as PCR and qPCR allow for the detection and quantification of phage DNA, with qPCR providing more sensitivity but unable to distinguish between infective and non-infective particles. Droplet Digital PCR (ddPCR) offers precise quantification, while mass spectrometry detects phage proteins. Next-Generation Sequencing (NGS) provides detailed genome data but is not ideal for quantification. In complex samples, specific pre-treatment steps are needed to enrich viral particles for accurate results. Each method has its advantages and limitations depending on the sample type and the level of accuracy required [117]. A summary of different methods for phage detection and quantification is shown in Table 3.

### Phage stability in the gastrointestinal tract

The primary challenge in administering phages orally is the acidic environment and proteolytic activity of the stomach, which can compromise phage viability [88]. Also, digestive enzymes can degrade phages before they reach the intestines. Research indicates that many phages are sensitive to acid and struggle to survive in the stomach’s acidic conditions. To enhance phage survival, Koo et al. suggested using antacids alongside vibriophages [118], while Smith et al. employed calcium carbonate (CaCO_3_) to shield phages from stomach acidity [119]. Similarly, Tanji’s phage cocktail was unstable at low pH, necessitating the use of CaCO_3_ for stabilization [120]. Another effective choice for the protection of phages against the destructive environment in the stomach is the microencapsulation of the phages [121].

**Table 3:** Methods of detection and quantification of phages

Method	Steps	Limitations
Plaque Counting (Golden Standard)	The DLA method is a standard technique for bacteriophage enumeration. It involves two layers of agar in a petri dish, a bottom layer supporting bacterial growth and a top layer containing both the bacterial host and a lower concentration of agar. Phages are added to the top agar, where they diffuse, infect the bacteria, and cause lysis, forming visible plaques. The number of plaques correlates to the phage titer, expressed as PFU [122].	This method requires optimization for each phage-host pair, and results can be affected by errors such as contamination, variations in bacterial growth, or operator bias.
**Detection of Whole Phage Particles**		
Transmission Electron Microscope (TEM)	TEM provides high-resolution images (up to 0.2 nm), enabling direct observation of phage particles [123].	It requires highly concentrated samples and is labor-intensive, expensive, and impractical for large-scale use.
Flow Cytometry	Flow cytometry detects viral particles marked with fluorescent dyes as they pass through a capillary. The technique is rapid and widely used and it is more suitable for distinguishing between different virus types in a sample [124].	It requires careful handling of samples to avoid interference from auto-fluorescence or contamination. It is also not directly linked to viral genome size.
NanoSight	NanoSight uses dynamic light scattering to enumerate phage particles by measuring how they scatter light. This technique is quick [125].	It requires high sample concentrations and clear samples, which can be challenging with complex sample types like soil or feces.
**Detection of Phage Nucleic Acids and Proteins**		
Polymerase Chain Reaction (PCR)	PCR detects phage nucleic acids, offering a faster alternative to plaque assays. Quantitative PCR (qPCR) further improves the method by allowing quantification of phage DNA, but its accuracy can be influenced by non-infectious phage-like particles [126].	The challenge lies in designing primers for diverse phages, as there is no universal gene present in all phages for easy detection.
Quantitative PCR (qPCR)	qPCR uses intercalating dyes or fluorescent probes to measure DNA quantities in real-time. It allows for rapid phage quantification, but results may differ from DLA due to the inclusion of non-infectious phage particles. qPCR can also be used to detect phages in clinical samples [125].	It cannot differentiate between viable and defective particles without additional treatment.
Droplet Digital PCR (ddPCR)	In ddPCR, the sample is partitioned into droplets, and each droplet undergoes PCR amplification, allowing accurate quantification without external standards. This method has been used to detect phages in various samples [127].	It may show discrepancies when compared to other methods like DLA.
Mass Spectrometry	Mass spectrometry detects and enumerates peptides derived from phage coat proteins [128].	It requires precise knowledge of protein copy numbers and is not yet suitable for mixed samples, as different phage mutations can affect results.
Next-Generation Sequencing (NGS)	NGS provides detailed information about phage genomes. Despite its limitations, NGS can provide semi-quantitative data about phage abundance. Single-molecule sequencing technologies (e.g., PacBio, Oxford Nanopore) offer improved performance and shorter run times, making them promising for phage detection and enumeration [129].	It is not ideal for quantifying phages due to the complexity of sequencing and assembling phage genomes.
Quantification in Complex Samples (clinical samples, feces, or food)	For sample preparation, methods like centrifugation, filtration, and concentration can enrich viral particles, improving detection accuracy [117].	Requires specific pre-treatment steps to remove inhibitors. Bioinformatics workflows for NGS require careful filtering of non-viral sequences to minimize contamination and obtain accurate phage counts.

### Phage delivery to the intestine

#### Effective delivery systems

Ensuring delivery of phages to the target site in sufficient quantities is a complex process due to several factors. For example, the acidic pH of the stomach is considered an important obstacle against successful phage therapy. It was reported that the co-administration of approved pharmacological agents that suppress gastric acid secretion, significantly improved phage survival and therapeutic efficacy within the gastrointestinal tract. In a single-blind, randomized, placebo-controlled clinical trial to evaluate the safety and feasibility of oral delivery of a two-phage cocktail in healthy adults, all participants were pretreated with oral esomeprazole to reduce gastric acidity to enhance phage survival through the gastrointestinal tract [130]. Another strategy includes encapsulation methods, like microencapsulation and liposome-based delivery, are being researched to protect phages from the harsh gastric environment. A study reported that liposome coating enhanced the retention of phages within the chicken intestinal tract. When both encapsulated and nonencapsulated phage cocktails were given to broilers, encapsulated phages were recovered from 38.1% of the birds after 72 h, compared to just 9.5% for nonencapsulated phages, indicating a significant difference in retention. Additionally, in vitro tests revealed that cecal contents from broilers facilitated the release of phages from the liposomes. In broilers infected with *Salmonella*, administering both phage types daily for six days provided comparable protection against colonization. However, the protective effect of nonencapsulated phages vanished once treatment ended, whereas the encapsulated phages continued to offer protection for at least a week, demonstrating their superior efficacy over time [131]. Another research study explored using alginate–carrageenan microcapsules with or without CaCO_3_ to protect *Salmonella* bacteriophages from harsh gastrointestinal conditions. While unprotected phages were inactivated at low pH, encapsulation improved phage survival, especially with added CaCO_3_, which further reduced phage loss. Phages were effectively released when the pH shifted to neutral, simulating the duodenum. The findings suggest that this encapsulation method could be a promising strategy for targeted phage delivery to the intestines [132].

### Microencapsulation of phages

Microencapsulation of phages refers to the process of enclosing bacteriophages in a protective coating or shell. This technique helps to safeguard the phages from environmental factors, improve their stability, and control their release, making them more effective. For instance, chitosan-alginate microencapsulation significantly improved the survival of Felix O1, a phage targeting *Salmonella*, in conditions mimicking the pig gastrointestinal tract [133]. Stanford et al. demonstrated that encapsulating phages rV5, wV7, wV8, and wV11 in methacrylate polymer effectively protected them from a pH of 3 in laboratory settings [134]. Another study used the orifice-coagulation bath method to microencapsulate phage T156, a novel *Salmonella* phage, that effectively inhibited *Salmonella* growth in in food matrices at both 4 °C and 25 °C. At 25 °C, the maximum antibacterial effectiveness observed was 57.93% in milk and 55.47% in lettuce [135]. A research study proved that the encapsulation of bacteriophages UAB_Phi20, UAB_Phi78, and UAB_Phi87 in liposomes increased the stability in a simulated gastric condition at pH 2.8. Nonencapsulated phages showed a significant titer decline of 5.7 to 7.8 log units, while encapsulated phages maintained greater stability, with reductions of only 3.7 to 5.4 log units [131]. Phage therapy for *Salmonella* infections was improved by microencapsulating phages using xanthan gum, sodium alginate, CaCl_2_, and chitooligosaccharides. This process enhanced phage stability, gastric acid resistance, and controlled release in the intestines. In experiments, microencapsulated phages showed better stability and therapeutic effects than free phages, making them a promising alternative for biological control of bacterial infections [136].

A recent study used microencapsulation to protect a two-phage cocktail containing *Salmonella* phage Epsilon15 and *Salmonella* phage SPN1S which was tested in vivo in a mouse model of oral *Salmonella* infection. A freeze-dried formulation of the phage cocktail, mixed with trehalose and whey protein, was created, yielding a phage titer of 10⁸ PFU/mL. Mice were treated with either the freeze-dried or free phage formula orally. Results showed that both forms of the phage cocktail effectively controlled MDR *Salmonella* infections, as demonstrated by histopathological improvements and weight gain in the animals. This formulation shows potential for clinical application in both humans and animals to treat MDR *Salmonella* infections [110].

### Pharmacokinetics of phage therapy (PT)

Pharmacokinetics refers to the body’s impact on drugs [137]. Lytic phages, unlike conventional antibiotics, are living biological entities that replicate in the presence of susceptible bacteria, while in the absence of a bacterial host, phage behaves similarly to nanoparticles, with their pharmacokinetics influenced by factors like size and surface features. However, when the phage interacts with its bacterial host, replication can occur, leading to more complex pharmacokinetic (PK) profiles involving absorption, distribution, metabolism, and elimination [138]. Pharmacokinetic parameters and different factors that influence these parameters are shown in Table 4. The key to determining their PK is the dose, which is measured in terms of phage particles. Quantifying phages is crucial but often overlooked, with plaque assays on agar plates being the most common method. However, plaque count may not accurately reflect the total number of phages, as only those capable of propagating and forming visible plaques are counted. The efficiency of plating, which measures the ratio of PFU on the target versus reference strain, must be considered. A lower efficiency of plating could result in an underestimation of phage concentration, requiring adjustment to account for this discrepancy [139].

Various delivery methods for phages, including oral, intravenous (IV) or intraperitoneal (IP), nasal, and topical routes, have been explored. While IV or IP administration ensures effective systemic delivery, the bioavailability of phages administered orally or nasally is often low. Nonetheless, these routes have shown promise for treating specific infections, such as gastrointestinal and respiratory infections. While oral delivery is effective for targeting gastrointestinal infections, it struggles with low blood absorption, even with high doses or acidity-neutralizing strategies. Topical delivery methods, like inhalation, are more efficient for localized infections, such as in the lungs, although biofilm barriers can hinder phage access to bacteria. Phages can reach organs like the liver and spleen quickly, but these organs may sequester phages, reducing their therapeutic impact. Other organs and tissues, including the brain, can also be penetrated by phages, but reaching effective concentrations remains challenging. To enhance phage therapy, molecular engineering, such as adding targeting peptides to phages, could improve their delivery to specific tissues and infection sites, thereby optimizing treatment outcomes [140].

**Table 4:** Pharmacokinetic parameters and different factors that influence these parameters

Pharmacokinetic parameter	Influencing Factors	The influence
Absorption	Size, shape, and the type of cells at the administration site	Smaller phages are more readily absorbed by cells, though the effect varies depending on the cell line.
Distribution	Physicochemical properties	After IV administration, phages are primarily accumulated in the spleen and liver, which are involved in filtering foreign particles.
	Microbiome	Bacteria with similar receptor sites may act as attractants for phages.
Metabolism	Host immunity (liver & spleen)	The liver’s Kupffer cells are particularly efficient in phagocytosing phages, while the spleen is thought to contribute to adaptive immunity by stimulating antibody production.
	Microbiome	Potentially affecting phage load in the body. Despite their specificity, phages have been shown to impact the gut microbiome, as evidenced by a reduction in *E. coli* after phage treatment in clinical trials.
	Stomach acidity (oral phages)	This effect may be reversible when the pH increases
Elimination	Size and renal excretion	Phages are poorly excreted renally due to their large particle size. Studies have shown significant variability in phage excretion rates after oral administration. These differences may be due to various factors, including the host’s age, health condition, type of infection, kidney function, and differences in how phages cross cellular barriers. Phage elimination profile is mainly determined by the ability to detect active phages through plaque assays.

### Pharmacodynamics of phage therapy

Pharmacodynamics (PD), refers to a drug’s impact on the body and its microbiota, considering both therapeutic benefits and potential side effects [140]. To reduce phage-associated side effects, it’s crucial to purify phage preparations by removing endotoxins and avoiding phages that may carry bacterial virulence factors. Strictly lytic phages tend not to carry these factors, which is beneficial for minimizing adverse effects. The effectiveness of phage therapy is largely influenced by phage host range and bacterial resistance to phages. Phage resistance, which can evolve, presents a challenge to their therapeutic potential [140].

The phage host range is an essential factor in determining the success of phage therapy. Phages can have different types of host ranges, a transductive host range, where phages transfer bacterial genes to a new host, where the genes can recombine with the host’s genome without killing the bacterial host [141]; a bactericidal host range, where phages kill bacteria without producing new virions; and a productive host range, where phages both kill bacteria and produce new virions [142]. Phage therapy can be passive, where phages reduce bacterial populations by sheer numbers, or active, where phages replicate in the presence of bacteria to propagate the phage population. Active therapy depends on several factors, including phage adsorption efficiency, burst size, and the ability of phages to replicate in the presence of bacteria. Passive treatments, on the other hand, rely on a high concentration of phages to kill bacteria, even if they do not replicate in situ. The immune system can also influence phage therapy. While immune responses may neutralize phages through antibodies, they can also enhance bacterial killing, acting as a “double-edged sword” [143].

Phage population growth is influenced by bacterial density and bacterial characteristics, such as physiological activity, which can affect phage replication. While passive treatments primarily rely on the presence of sufficient phage numbers, the ability of phages to replicate in vivo is beneficial for maximizing their effectiveness. To address the limitations of single-phage treatments, phage cocktails are often employed. These cocktails combine different phages to broaden the host range and increase the likelihood of successful treatment [143]. Bacterial resistance to phages is also an obstacle to effective phage therapy. Resistance mechanisms can be innate, such as blocks to phage adsorption or penetration, or adaptive, such as clustered regularly interspaced short palindromic repeats- CRISPR-associated protein (CRISPR-Cas systems) [144]. Resistance can develop before treatment (community resistance) or during treatment (treatment resistance). Community resistance can be managed through regular monitoring of bacterial resistance patterns and updating phage cocktails accordingly. In contrast, treatment resistance may arise during therapy, but phage cocktails, which target multiple bacterial strains, can help delay resistance development [140].

Phage pharmacodynamics also faces challenges due to the absence of standardized methods to assess phage antibacterial activity. Traditional testing methods, like the direct spot test, efficiency of plating, and planktonic killing assays, each have strengths and limitations. These discrepancies highlight the need for standardized PD parameters to optimize phage therapy. MOI refers to the ratio of phages to bacteria, plays a critical role in resistance development and phage therapy effectiveness. Higher MOI may prevent bacterial resistance but could also lead to mutations that foster resistance, underscoring the need to understand the optimal MOI for phage therapy [140]. The PK and PD of phages are influenced by the infection environment. For example, biofilms can act as a significant barrier to phage action, although some phages are capable of breaking down biofilms and improving treatment efficacy. Unlike antibiotics, which have fixed PK/PD relationships, phage therapy follows a more dynamic “predator-prey” model, where phages replicate in response to bacterial presence, creating a complex PK/PD scenario. Phages often accumulate in infected tissues, and their PK can vary based on infection conditions [139]. Mathematical models have been developed to better understand phage dynamics, including their interaction with bacteria and the immune system, which can help optimize phage use. However, clinical application of these models remains limited due to insufficient data on phage-bacteria-immune system interactions. Clinical trials of phage therapy have shown mixed results, highlighting the need for further research into phage PK/PD, bacterial concentrations, immune responses, and the role of phage-neutralizing antibodies. Standardized clinical trials with detailed monitoring are necessary to advance phage therapy in clinical settings [145].

### Phage-bacteria interactions

#### Resistance development

Bacteria, like other genetic entities, can evolve in response to environmental changes, including exposure to chemotherapies such as antibiotics or phages [146]. Resistance to phages can be inherent in certain bacterial species or acquired over time. Acquired resistance can either emerge before exposure to the antagonist or as a result of selective pressures during treatment [140]. Resistance may be horizontal, arising through the transfer of mobile genetic elements, or mutational, involving changes to bacterial molecules that phages need to infect successfully [147]. Bacteria can also develop either phenotypic tolerance (temporary resistance) or genetic resistance (stable changes). Mutations that lead to phage resistance can cause antagonistic pleiotropies, meaning that while resistance provides benefits, it may also come with costs, such as slower growth, reduced virulence, or greater susceptibility to antibiotics [148]. These trade-offs may decrease the bacteria’s ability to cause infections [149].

Resistance to phages has been noted in research analyzing clinical samples from patients infected with *V. cholerae*, as well as in phage-treated chickens infected with *C. jejuni* and calves infected with *E. coli* [150–152]. This requires continuous monitoring and development of phage cocktails. It is advisable to utilize a cocktail of phages with varying host ranges and receptor targets in all phage therapy studies to enhance effectiveness and minimize the development of phage resistance in targeted pathogens [153]. Phage cocktails are generally less toxic and can be safely used regularly compared to untested antibiotic combinations, which might cause unpredictable side effects [154].

#### Biofilm formation

Some bacterial strains can form biofilms, which are protective layers that phages find difficult to penetrate. Recent research has shown that bacteria have developed several defense mechanisms to survive phage infections, including surface modification, superinfection exclusion (Sie), restriction-modification (R-M) systems, CRISPR-Cas systems, and abortive infection (Abi) systems [155]. Surface modification prevents phages from attaching to bacterial cells, making it one of the safest defenses. For example, glycosylated type IV pili can block phage replication and protect *P. aeruginosa* from pilus-specific phages [156]. Sie prevents secondary infections by a phage when the bacterium is already infected [157], allowing bacteria to resist further attacks from similar or closely related phages [158]. The R-M and CRISPR-Cas systems are common and diverse bacterial defenses. R-M systems, which include restriction endonucleases and methyltransferases, cut phage DNA that is not properly modified, while protecting the bacterial genome through methylation [159]. Bacteria can also enhance their phage resistance by increasing the concentration of restriction enzymes [160]. CRISPR-Cas systems, an adaptive immune mechanism, target and cut foreign DNA from phages, preserving the bacterial genome’s integrity [161].

Abi systems represent a final defense where infected bacteria undergo “altruistic suicide,” halting phage spread and protecting the population [162]. These systems prevent phage replication by interfering with its lifecycle after adsorption and DNA injection, resulting in few or no infectious virions and the death of the infected cells [163]. In response to bacterial resistance to PT through surface modifications, mutations, and modified restriction enzymes, phage cocktails, which combine multiple phages, overcome this developing resistance, by broadening host range and reducing resistance [164]. Studies demonstrated that, they are more effective at eradicating bacterial biofilms compared to single-phage treatments [165, 166]. Also, combining phages with antibiotics has been shown to enhance bacterial killing and improve treatment efficacy [167]. Phages can weaken biofilm structures, making bacteria more susceptible to antibiotics when administered in the right sequence [168, 169].

Furthermore, genetically engineered phages are being developed to target a broader range of bacteria and enhance biofilm degradation [170]. These modified phages can improve biofilm removal and bacterial elimination [171–173].

Last but not least, phage-derived enzymes, such as depolymerases, lysins, and DNases, are also effective in breaking down biofilms and facilitating bacterial elimination [174]. These enzymes can be used alone or as adjuvants for better biofilm eradication [175, 176]. Finally, phages can be combined with chemical disinfectants or nanomaterials to enhance biofilm removal and penetration [177, 178]. These strategies improve phage delivery to biofilm layers, increasing their effectiveness in treating infections.

#### Bacteriophages induced gene transfer

Bacteriophages contribute to horizontal gene transfer primarily through transduction, a process by which bacterial DNA is inadvertently packaged into phage particles and delivered to new host cells. This occurs either randomly during the lytic cycle (generalized transduction) or specifically during excision of temperate phages from the host genome (specialized transduction). Although this mechanism raises concerns about the spread of antibiotic resistance and virulence factors, recent genomic analyses indicate that such genes are rarely found in therapeutic phages. Additionally, while functional assays have detected transduction events in laboratory settings, these may overestimate the actual clinical risk. Importantly, gene transfer potential depends more on the phage’s DNA packaging system than on its lifecycle, suggesting that not all temperate phages pose a high risk [179]. Therefore, risk assessments for phage therapy should be based on detailed genetic and mechanistic evaluations rather than generalized exclusions.

### Regulatory and safety concerns

#### Regulatory approval

The regulatory pathway for phage therapy is still developing, with different standards across countries, making global approval challenging [180–182]. PT involves single phages or “phage cocktails” and can be tailored to an individual patient’s bacterial infection, offering a personalized approach. However, only a few centers worldwide, including those in Georgia, Belgium, Poland, the USA, and Australia, continue to provide phage-based treatments and conduct clinical trials on their effectiveness [183–185]. Phage therapy has been used experimentally for decades at institutions like the Eliava Institute in Georgia and the Ludwik Hirschweld Institute in Poland. It follows the principles of the Helsinki Declaration and is intended for patients for whom no proven medical treatments exist or have been ineffective [181, 186–189]. Following Poland’s EU accession in 2005, its PT became a global model, supported by the establishment of the Phage Therapy Center (PTC), which operates in compliance with EU and US regulations [190]. Belgium introduced regulations for individual patient treatments with personalized phage preparations in 2018, making it one of the few Western countries to embrace PT [191]. That same year, the Center for Innovative Phage Applications and Therapeutics (IPATH) was established at the University of California, San Diego, focusing on PT for life-threatening infections and incorporating clinical trials [192]. Australia, with its favorable regulatory environment, is well-suited for conducting controlled phage trials, with the Therapeutic Goods Administration (TGA) aligning with European Medicines Agency (EMA) policies [193]. The therapeutic use of bacteriophages in the UK is regulated under the Human Medicines Regulations 2012, with oversight by the Medicines and Healthcare products Regulatory Agency (MHRA), which treats phages as biological medicinal products requiring compliance with quality, safety, and efficacy standards. While personalized phage therapies can be administered under the “specials” exemption for individual cases, they remain outside routine licensing pathways and must still adhere to Good Manufacturing Practice (GMP) standards. Key regulatory challenges include ensuring product consistency, sterility, and generating clinical evidence to support broader use. The current framework does not yet fully accommodate the adaptive and personalized nature of phage therapy, highlighting the need for further regulatory refinement [194].

Phage banks, which hold extensive collections of phages, play a crucial role in matching the appropriate phage to a bacterial infection. Notable collections include the American Type Culture Collection (ATCC) and the Public Health England (PHE). Creative Biolabs, a biopharmaceutical company, aids in developing PT by screening natural phages and engineering synthetic ones targeting specific bacteria [195]. The rise in MDR organisms has driven the need for expanding phage collections. The global market for phage therapy is projected to reach USD 1.4 billion by 2026 [195]. Advancements in the production and purification of phages are expected to further propel the development of PT in Western Europe and beyond [196].

### Safety

#### Host immune responses

PT holds significant therapeutic promise but faces safety concerns that have limited its clinical use. One primary concern is the presence of endotoxins in phage preparations. These endotoxins, LPS from Gram-negative bacteria, are released when bacteria divide or die and can provoke severe immune reactions, including fever, reduction in white blood cells, and even shock. To mitigate these risks, the U.S. FDA mandates that LPS levels in intravenously administered biologics be below 5 Endotoxin Units per kilogram of body weight, requiring improvements in endotoxin removal methods for PT to meet these safety standards [197]. Another concern is the potential production of antibodies against phages. Immunoglobulins like IgM and IgG can neutralize or diminish the effectiveness of phages by triggering their rapid clearance from the bloodstream [198]. Additionally, phages consist of proteins and nucleic acids that may induce immune responses, including allergic reactions. While these immune responses are typically mild, caution is still necessary [199].

#### Phage effect on human microbiota

Phages may also transfer virulence or antibiotic resistance genes to host bacteria, particularly during the lysogeny process, where phage genetic material is integrated into bacterial genomes [200]. This could increase the pathogenicity of bacteria within the normal human microbiota, creating potential health risks. Moreover, the use of broad-spectrum phages could disrupt the natural microbiota, which may have unintended consequences [201]. Phages can also evolve, potentially losing their ability to kill bacteria during manufacture or use, which poses further challenges to their effectiveness [202]. However, targeted manipulation of bacterial communities using engineered bacteriophages can be used to allow phage-delivered genome editing for microbiome modulation. In parallel to the prompt advancement in molecular biology, bioinformatics, and gene-editing technologies, engineered phages grant an effective strategy for targeting harmful genes in the gut microbiota without distracting the integrity of the microbiome, thus presenting potential antimicrobial therapy. Phage-based engineering of the gut microbiome aims to genetically modify bacterial pathogens and consequently eradicate them. This treatment strategy relies on the application of engineered phages to treat diarrhea caused by *Escherichia Coli*, *Clostridium difficile* infection, as well as colorectal cancer due to *Fusobacterium nucleatum* infection [203].

#### Possible solutions to ensure the safety of phage therapy

To ensure the safety and efficacy of PT, it is important to select phages that are highly effective against bacteria while being non-toxic to the patient [204]. Phage preparations must be carefully characterized, ideally through whole genome sequencing, to exclude any harmful genes. Purifying phages can also minimize the risks of allergic or toxic reactions [201]. Despite these advancements, the complex and costly process of producing phage preparations at scale remains a challenge, limiting their widespread use in clinical settings. However, technological progress may eventually make large-scale clinical applications of PT possible. Phages are specific to bacteria and do not replicate in mammalian cells. However, mammalian cells can internalize phages through processes like macropinocytosis and receptor-mediated endocytosis [205]. Some phages can bind to specific cell receptors, facilitating their entry into mammalian cells. While phages are generally considered safe for human use, studies have shown that they can stimulate immune responses and even enhance cell growth and metabolism [206]. Phages are also being explored for their potential as adjuvants or delivery systems for vaccines [207]. Moreover, Bacteriophage-derived endolysins are emerging as superior alternatives to whole bacteriophage therapy due to their immediate, targeted lytic action, absence of genetic material, and lower risk of horizontal gene transfer or unintended ecological impact [208]. Unlike whole phages that require bacterial replication and may trigger immune responses or carry resistance genes, lysins act directly on bacterial cell walls, offering precise and rapid antimicrobial effects, especially against Gram-positive pathogens [209]. Recent advances in encapsulation techniques—using polymers, liposomes, nanofibers, and stimuli-responsive materials—have significantly enhanced lysin stability, bioavailability, and targeted delivery in harsh physiological environments [210]. These systems allow lysins to maintain activity across various applications, including gut infections, chronic wounds, and foodborne pathogen control [208]. In contrast, while whole phages are advantageous in dynamic microbial ecosystems due to their self-replicating nature and biofilm penetration capabilities, their unpredictable pharmacokinetics and regulatory hurdles limit their broader use [209]. Therefore, lysins, especially when encapsulated, offer a safer, more tunable, and clinically viable antimicrobial strategy for precision-targeted infection control [210]. Although PT has shown promise, the interactions between phages and mammalian cells remain poorly understood, and further research is needed to fully assess the implications of these interactions. A better understanding of how phages interact with the human immune system and cells will be essential for optimizing their use in clinical treatments and ensuring their safety.

## Conclusion

Phage therapy represents a promising alternative to conventional antimicrobials for the treatment of gastrointestinal-associated infections. However, there are still critical challenges that must be addressed before its widespread clinical application. These challenges include difficulties related to phage identification and isolation, bacterial host resistance to phages, and the inconsistencies in the pharmacokinetics and pharmacodynamics of the phage preparations. Moreover, there are considerable legal obstacles, such as the need to standardize phage formulations and the requirement for extensive clinical studies. Despite these challenges, numerous successful case reports support the optimism generated by combining and optimizing existing phage therapies for the treatment of infections with increased antimicrobial resistance. Future studies should concentrate on overcoming the constraints of phage stability, adaptability, specificity, immune response regulation, and ethical issues to enable closer alignment between phage therapy and general clinical practice.

## Data Availability

No datasets were generated or analysed during the current study.

## Abbreviations

AEs: Adverse events
AMR: Antimicrobial resistance
ATCC: American type culture collection
Cas: CRISPR-associated protein
C. coli: Campylobacter coli
C. difficile: Clostridium difficile
C. jejuni: Campylobacter jejuni
CDC: Centers for disease control and prevention
CFU: Colony-forming unit
CRC: Colorectal cancer
CRISPR: Clustered regularly interspaced short palindromic repeats
ddPCR: Droplet digital PCR
DLA: Double agar overlay
E. coli: Escherichia coli
EMA: European medicines agency
EPS: Extracellular polymeric substances
ESBLs: Extended-spectrum β-lactamase producers
ETEC: Enterotoxigenic escherichia coli
EU: European union
GI: Gastrointestinal
H. pylori: Helicobacter pylori
IP: Intraperitoneal
IPATH: Innovative phage applications and therapeutics
IV: Intravenous
KPC-2: Klebsiella pneumoniae carbapenemase-2
MDR: Multidrug-resistant
MOI: Multiplicities of infection
NDM: New delhi metallo-beta-lactamase 1
NGS: Next-generation sequencing
NTS: Non-typhoidal salmonella
OPT: Oral phage therapy
OXA-48-like: Oxacillinase-48-like enzymes
PCR: Polymerase chain reaction
PD: Pharmacodynamics
PFU: Plaque forming unit
PHE: Public health england
PK: Pharmacokinetic
PT: Phage therapy
PTC: Phage therapy center
Qpcr: Quantitative PCR
STEC: Shiga toxin-producing E. coli
TEM: Transmission electron microscopy
TGA: Therapeutic goods administration
TS: Typhoidal Salmonella
US: United States
XDR: Extensively drug-resistant
Y. enterocolitica: Yersinia enterocolitica

## References

[1] Liang D, et al. Global incidence of diarrheal Diseases—An update using an interpretable predictive model based on XGBoost and SHAP: A systematic analysis. Nutrients. 2024;16(18):3217. 39339819 10.3390/nu16183217 PMC11434730

[2] Ranasinghe S, Fhogartaigh CN. Bacterial gastroenteritis. Medicine. 2021;49(11):687–93.

[3] Holst MM. Contributing factors of foodborne illness outbreaks—National outbreak reporting system, united states, 2014–2022. MMWR Surveillance Summaries, 2025 74(1):1-12. 10.15585/mmwr.ss7401a1 PMC11908744 40067777

[4] Subramanian A. Emerging roles of bacteriophage-based therapeutics in combating antibiotic resistance. Front Microbiol. 2024;15:1384164. 39035437 10.3389/fmicb.2024.1384164 PMC11257900

[5] Cui L et al. A comprehensive review on phage therapy and phage-Based drug development. Antibiot (Basel), 2024;13(9). 10.3390/antibiotics13090870 PMC11428490 39335043

[6] Mayorga-Ramos A, et al. Bacteriophage-mediated approaches for biofilm control. Front Cell Infect Microbiol. 2024;14:1428637. 39435185 10.3389/fcimb.2024.1428637 PMC11491440

[7] Supina BSI, Dennis JJ. The current landscape of Phage–Antibiotic synergistic (PAS) interactions. Antibiotics. 2025;14(6):545. 40558135 10.3390/antibiotics14060545 PMC12190067

[8] Liu C et al. Phage-Antibiotic therapy as a promising strategy to combat Multidrug-Resistant infections and to enhance antimicrobial efficiency. Antibiot (Basel), 2022;11(5). 10.3390/antibiotics11050570 PMC9137994 35625214

[9] Enciso-Martínez Y, et al. Relevance of tracking the diversity of Escherichia coli pathotypes to reinforce food safety. Int J Food Microbiol. 2022;374:109736. 35613497 10.1016/j.ijfoodmicro.2022.109736

[10] WHO). W.H.O., Diarrhoeal disease. 2024.

[11] SULEIMAN K, et al. Bacterial diarrhea among infants in developing countries: an overview of diarrheagenic Escherichia coli (DEC). Gadau J Pure Allied Sci. 2022;1(1):73–81.

[12] Alum EU, Obeagu EI, Ugwu O. Curbing diarrhea in children below five years old: the sub-Saharan African standpoint. Volume 5. J. New Medical Innovations and Research; 2024;1.

[13] Walker L, Sun S, Thippareddi H. Growth comparison and model validation for growth of Shiga toxin-producing Escherichia coli (STEC) in ground beef. LWT. 2023;182:114823.

[14] Khairy RMM, et al. Prevalence, phylogeny, and antimicrobial resistance of Escherichia coli pathotypes isolated from children less than 5 years old with community acquired- diarrhea in upper Egypt. BMC Infect Dis. 2020;20(1):908. 33256619 10.1186/s12879-020-05664-6 PMC7708180

[15] Mansour A, et al. Diarrhea burden due to natural infection with enterotoxigenic Escherichia coli in a birth cohort in a rural Egyptian community. J Clin Microbiol. 2014;52(7):2595–603. 24829232 10.1128/JCM.00215-14 PMC4097688

[16] Grudlewska-Buda K, et al. Antibiotic resistance in selected emerging bacterial foodborne Pathogens—An issue of concern?? Antibiotics. 2023;12(5):880. 37237783 10.3390/antibiotics12050880 PMC10215942

[17] Sivakumar M, et al. Extended-spectrum beta-lactamase (ESBL) producing and multidrug-resistant Escherichia coli in street foods: a public health concern. J Food Sci Technol. 2021;58:1247–61. 33746253 10.1007/s13197-020-04634-9 PMC7925767

[18] Nasrollahian S, Graham JP, Halaji M. A review of the mechanisms that confer antibiotic resistance in pathotypes of E. coli. Front Cell Infect Microbiol, 2024; 14:1387497. 10.3389/fcimb.2024.1387497 PMC11024256 38638826

[19] Ayuti SR, et al. Tackling salmonellosis: A comprehensive exploration of risks factors, impacts, and solutions. Open Vet J. 2024;14(6):1313–29. 39055762 10.5455/OVJ.2024.v14.i6.1 PMC11268913

[20] Lamichhane B, et al. Salmonellosis: an overview of epidemiology, pathogenesis, and innovative approaches to mitigate the antimicrobial resistant infections. Antibiotics. 2024;13(1):76. 38247636 10.3390/antibiotics13010076 PMC10812683

[21] Ehuwa O, Jaiswal AK, Jaiswal S. Salmonella, food safety and food handling practices. Foods. 2021;10(5):907. 33919142 10.3390/foods10050907 PMC8143179

[22] Delahoy MJ, et al. Preliminary incidence and trends of infections caused by pathogens transmitted commonly through Food - Foodborne diseases active surveillance network, 10 U.S. Sites, 2022. MMWR Morb Mortal Wkly Rep. 2023;72(26):701–6. 37384552 10.15585/mmwr.mm7226a1 PMC10328488

[23] Wang Z, et al. Nationwide trends and features of human salmonellosis outbreaks in China. Emerg Microbes Infections. 2024;13(1):2372364. 10.1080/22221751.2024.2372364 PMC11259058 38923510

[24] Patel P, et al. Updates and current knowledge on the common forms of gastroenteritis: A review. J Clin Med. 2025;14(10):3465. 40429459 10.3390/jcm14103465 PMC12112049

[25] Youssef RA, et al. Serotyping and antimicrobial resistance profile of enteric nontyphoidal Salmonella recovered from febrile neutropenic patients and poultry in Egypt. Antibiotics. 2021;10(5):493. 33925773 10.3390/antibiotics10050493 PMC8147078

[26] Zeng S, et al. Prevalence of chromosomally located Bla CTX-M-55 in Salmonella typhimurium ST34 isolates recovered from a tertiary hospital in guangzhou, China. Microbiol Spectr. 2022;10(3):e02771–21. 35616373 10.1128/spectrum.02771-21 PMC9241639

[27] Nadi ZR, et al. Evaluation of antibiotic resistance and prevalence of common Salmonella enterica serovars isolated from foodborne outbreaks. Microchem J. 2020;155:104660.

[28] Chang Y-J, et al. Changing antimicrobial resistance and epidemiology of non-typhoidal Salmonella infection in Taiwanese children. Front Microbiol. 2021;12:648008. 33868207 10.3389/fmicb.2021.648008 PMC8044818

[29] Ferrari RG, et al. Worldwide epidemiology of Salmonella serovars in animal-based foods: a meta-analysis. Appl Environ Microbiol. 2019;85(14):e00591–19. 31053586 10.1128/AEM.00591-19 PMC6606869

[30] Britto CD, et al. A systematic review of antimicrobial resistance in Salmonella enterica serovar typhi, the etiological agent of typhoid. PLoS Negl Trop Dis. 2018;12(10):e0006779. 30307935 10.1371/journal.pntd.0006779 PMC6198998

[31] Levine MM, Simon R. The gathering storm: is untreatable typhoid fever on the way? MBio. 2018;9(2):pmbio101128–00482. 10.1128/mBio.00482-18 PMC5874914 29559573

[32] Shah SAA et al. Antimicrobial sensitivity pattern of Salmonella typhi: emergence of resistant strains. Cureus, 2020;12(11). 10.7759/cureus.11778 PMC7779132 33409025

[33] Chatham-Stephens K. Emergence of extensively drug-resistant Salmonella Typhi infections among travelers to or from Pakistan—United states, 2016–2018. MMWR. Morbidity and Mortality Weekly Report; 2019;68. 10.15585/mmwr.mm6801a3 PMC6342547 30629573

[34] Hooda Y, et al. Molecular mechanism of Azithromycin resistance among typhoidal Salmonella strains in Bangladesh identified through passive pediatric surveillance. PLoS Negl Trop Dis. 2019;13(11):e0007868. 31730615 10.1371/journal.pntd.0007868 PMC6881056

[35] Safdar N, et al. Pan Drug-Resistant Salmonella serovar Typhi septicaemia in A child: A case report. J Pak Med Assoc. 2023;73(9):1909–11. 37817712 10.47391/JPMA.8154

[36] Punchihewage-Don AJ, Ranaweera PN, Parveen S. Defense mechanisms of Salmonella against antibiotics: a review. Front Antibiot, 2024.;3:1448796. 10.3389/frabi.2024.1448796 PMC11731628 39816264

[37] Peh E, et al. Bacteriophage cocktail application for Campylobacter mitigation-from in vitro to in vivo. BMC Microbiol. 2023;23(1):209. 37543585 10.1186/s12866-023-02963-1 PMC10403930

[38] Janež N, Loc-Carrillo C. Use of phages to control Campylobacter spp. J Microbiol Methods. 2013;95(1):68–75. 23830848 10.1016/j.mimet.2013.06.024

[39] European Food Safety Authority (EFSA); European Centre for Disease Prevention and Control (ECDC). The European Union One Health 2022 Zoonoses Report. EFSA J. 2023;21(12):e8442. 38089471 10.2903/j.efsa.2023.8442 PMC10714251

[40] Maraki S, et al. Prevalence and antimicrobial resistance of bacterial enteropathogens in crete, greece, during 2011–2022. Acta Microbiol Immunol Hung. 2024;71(1):69–75. 38345622 10.1556/030.2024.02214

[41] Bunduruș IA, et al. Overview of virulence and antibiotic resistance in Campylobacter spp. Livest Isolates Antibiot. 2023;12(2):402. 10.3390/antibiotics12020402 PMC9952398 36830312

[42] Nisa I, et al. Shigella flexneri: an emerging pathogen. Folia Microbiol. 2020;65(2):275–91. 32026288 10.1007/s12223-020-00773-w

[43] Salleh MZ, et al. Prevalence of Multidrug-Resistant and Extended-Spectrum Beta-Lactamase-Producing Shigella species in asia: A systematic review and Meta-Analysis. Antibiotics. 2022;11(11):1653. 36421297 10.3390/antibiotics11111653 PMC9687025

[44] Shad AA, Shad WA. Shigella sonnei: virulence and antibiotic resistance. Arch Microbiol. 2021;203(1):45–58. 32929595 10.1007/s00203-020-02034-3 PMC7489455

[45] Ahamed ST, Giria N. Shigellosis and development of multiple antimicrobial resistance mechanisms of Shigella spp. Biosci Biotechnol Res Asia. 2021;18(4):703–18.

[46] Pakbin B, Brück WM, Brück TB. Molecular mechanisms of Shigella pathogenesis; recent advances. Int J Mol Sci. 2023;24(3):2448. 36768771 10.3390/ijms24032448 PMC9917014

[47] Feuerstadt P, Theriault N, Tillotson G. The burden of CDI in the united states: a multifactorial challenge. BMC Infect Dis. 2023;23(1):132. 36882700 10.1186/s12879-023-08096-0 PMC9990004

[48] Principi N, et al. Prevention of clostridium difficile infection and associated diarrhea: an unsolved problem. Microorganisms. 2020;8(11):1640. 33114040 10.3390/microorganisms8111640 PMC7690700

[49] Budayanti NNS, Aryana IGPS, Wedari NLPH. Clostridium diffi cile infection (CDI) by hypervirulent BI/NAP1/027 strain: a comprehensive review of toxigenicity, pathogenesis, risk factors, and preventative measures. Indonesian J Trop Infect Disease. 2022;10(1):27–41.

[50] Wickramage I, Spigaglia P, Sun X. Mechanisms of antibiotic resistance of clostridioides difficile. J Antimicrob Chemother. 2021;76(12):3077–90. 34297842 10.1093/jac/dkab231 PMC8598299

[51] Contreras-Omaña R, Escorcia-Saucedo AE, Velarde-Ruiz JA, Velasco. Prevalence and impact of antimicrobial resistance in Gastrointestinal infections: A review. Revista De Gastroenterología De México (English Edition). 2021;86(3):265–75. 10.1016/j.rgmxen.2021.06.004 34158260

[52] Martínez-Meléndez A, et al. Circulation of highly drug-resistant clostridium difficile ribotypes 027 and 001 in two tertiary-care hospitals in Mexico. Microb Drug Resist. 2018;24(4):386–92. 29485939 10.1089/mdr.2017.0323

[53] Pal M, et al. Staphylococcus aureus: A major pathogen of food poisoning: A rare research report. Nutr Food Process. 2022;5(1):1–3.

[54] Gherardi G. Staphylococcus aureus infection: pathogenesis and antimicrobial resistance. Int J Mol Sci. 2023;24(9):8182. 37175886 10.3390/ijms24098182 PMC10179453

[55] Lee D, et al. Expression of cholera toxin (CT) and the toxin Co-Regulated Pilus (TCP) by variants of ToxT in vibrio cholerae strains. Toxins. 2023;15(8):507. 37624264 10.3390/toxins15080507 PMC10467113

[56] Asantewaa AA, et al. Cholera outbreaks in Low- and Middle-Income countries in the last decade: A systematic review and Meta-Analysis. Microorganisms. 2024;12(12):2504. 39770707 10.3390/microorganisms12122504 PMC11728267

[57] De R. Mobile genetic elements of vibrio cholerae and the evolution of its antimicrobial resistance. Front Trop Dis, 2021.;2:691604.

[58] Vashist A, et al. Molecular insights into genomic Islands and evolution of vibrio cholerae, in microbial genomic Islands in adaptation and pathogenicity. Springer; 2023;279–324.

[59] Jubyda FT, et al. Vibrio cholerae O1 associated with recent endemic cholera shows Temporal changes in serotype, genotype, and drug-resistance patterns in Bangladesh. Gut Pathogens. 2023;15(1):17. 37046358 10.1186/s13099-023-00537-0 PMC10090749

[60] Riahi SM, Ahmadi E, Zeinali T. Global prevalence of yersinia Enterocolitica in cases of gastroenteritis: A systematic review and meta-analysis. Int J Microbiol. 2021;2021(1):1499869. 34512763 10.1155/2021/1499869 PMC8433020

[61] Grygiel-Górniak B. Current challenges in yersinia diagnosis and treatment. Microorganisms. 2025;13(5):1133. 40431305 10.3390/microorganisms13051133 PMC12114158

[62] European Food Safety Authority (EFSA); European Centre for Disease Prevention and Control (ECDC). The European Union One Health 2022 Zoonoses Report. EFSA J. 2023 Dec 12;21(12):e8442. 38089471 10.2903/j.efsa.2023.8442 PMC10714251

[63] Huang C, Li W, Chen J. Stringent response factor DksA contributes to fatty acid degradation function to influence cell membrane stability and polymyxin B resistance of yersinia Enterocolitica. Int J Mol Sci. 2023;24(15):11951. 37569327 10.3390/ijms241511951 PMC10418728

[64] Karlsson PA, et al. Molecular characterization of multidrug-resistant yersinia Enterocolitica from foodborne outbreaks in Sweden. Front Microbiol. 2021;12:664665. 34054769 10.3389/fmicb.2021.664665 PMC8155512

[65] Engelsberger V, Gerhard M, Mejías-Luque R. Effects of Helicobacter pylori infection on intestinal microbiota, immunity and colorectal cancer risk. Front Cell Infect Microbiol. 2024;14:1339750. 38343887 10.3389/fcimb.2024.1339750 PMC10853882

[66] Chivu R, et al. Navigating through surgical implications of: an Up-to-Date comprehensive literature review. Chirurgia. 2023;118:568–83. 38228590 10.21614/chirurgia.2023.v.118.i.6.p.568

[67] Lin Y, et al. Antibiotic resistance in Helicobacter pylori: from potential biomolecular mechanisms to clinical practice. J Clin Lab Anal. 2023;37(7):e24885. 37088871 10.1002/jcla.24885 PMC10220298

[68] Srisuphanunt M, et al. Molecular mechanisms of antibiotic resistance and novel treatment strategies for Helicobacter pylori infections. Trop Med Infect Disease. 2023;8(3):163. 36977164 10.3390/tropicalmed8030163 PMC10057134

[69] Pessoa RBG et al. Aeromonas and human health disorders: clinical approaches. Front Microbiol, 2022;13:868890 10.3389/fmicb.2022.868890 PMC9195132 35711774

[70] Cao B, Yan J, Santos JA, et al. Chap. 50 - Plesiomonas. Molecular medical microbiology (Third Edition), Y.-W. Tang. Academic; 2024;1027–42.

[71] Zhang P, et al. Pathogenic characteristics of an aggregated diarrhea event caused by Plesiomonas shigelloides from stream. PLoS ONE. 2024;19(4):e0301623. 38574097 10.1371/journal.pone.0301623 PMC10994385

[72] Janda JM, Duman M. Expanding the spectrum of diseases and disease associations caused by Edwardsiella Tarda and related species. Microorganisms. 2024;12(5):1031. 38792860 10.3390/microorganisms12051031 PMC11124366

[73] Zhou Y, et al. A case of septic shock caused by drug-resistant Edwardsiella Tarda and literature review. BMC Infect Dis. 2025;25(1):393. 40119266 10.1186/s12879-025-10789-7 PMC11929179

[74] Clokie MR, et al. Phages in nature. Bacteriophage. 2011;1(1):31–45. 21687533 10.4161/bact.1.1.14942 PMC3109452

[75] Gamachu SB, Debalo M. Review of bacteriophage and its applications. Int J Veterinary Sci Res. 2022;8(3):133–47.

[76] Jordá J, et al. Phage-Based biosanitation strategies for minimizing persistent Salmonella and Campylobacter bacteria in poultry. Animals. 2023;13(24):3826. 38136863 10.3390/ani13243826 PMC10740442

[77] Gordillo Altamirano FL, Barr JJ. Phage therapy in the postantibiotic era. Clin Microbiol Rev. 2019;32(2). 10.1128/cmr. 00066– 18. 10.1128/CMR.00066-18 PMC6431132 30651225

[78] Durr HA, Leipzig ND. Advancements in bacteriophage therapies and delivery for bacterial infection. Mater Adv. 2023;4(5):1249–57. 36895585 10.1039/d2ma00980c PMC9987412

[79] Ali Y, et al. The current status of phage therapy and its advancement towards Establishing standard antimicrobials for combating multi drug-resistant bacterial pathogens. Microb Pathog. 2023;181:106199. 37336428 10.1016/j.micpath.2023.106199

[80] Flemming H-C, et al. Biofilms: an emergent form of bacterial life. Nat Rev Microbiol. 2016;14(9):563–75. 27510863 10.1038/nrmicro.2016.94

[81] Vila MM, Balcão LM, Balcão VM. Phage delivery strategies for biocontrolling human, animal, and plant bacterial infections: state of the Art. Pharmaceutics. 2024;16(3):374. 38543268 10.3390/pharmaceutics16030374 PMC10976114

[82] Ryan EM, et al. Recent advances in bacteriophage therapy: how delivery routes, formulation, concentration and timing influence the success of phage therapy. J Pharm Pharmacol. 2011;63(10):1253–64. 21899540 10.1111/j.2042-7158.2011.01324.x

[83] Al-Anany AM, et al. Phage therapy in the management of urinary tract infections: a comprehensive systematic review. Phage. 2023;4(3):112–27. 37771568 10.1089/phage.2023.0024 PMC10523411

[84] Bruttin A, Brüssow H. Human volunteers receiving Escherichia coli phage T4 orally: a safety test of phage therapy. Antimicrob Agents Chemother. 2005;49(7):2874–8. 15980363 10.1128/AAC.49.7.2874-2878.2005 PMC1168693

[85] Johri AV, et al. Case report: chronic bacterial prostatitis treated with phage therapy after multiple failed antibiotic treatments. Front Pharmacol. 2021;12:692614. 34177601 10.3389/fphar.2021.692614 PMC8222915

[86] Leszczyński P, et al. Successful eradication of methicillin-resistant Staphylococcus aureus (MRSA) intestinal carrier status in a healthcare worker—case report. Folia Microbiol. 2006;51:236–8. 17004656 10.1007/BF02932128

[87] Mao X, et al. Oral phage therapy with microencapsulated phage A221 against Escherichia coli infections in weaned piglets. BMC Vet Res. 2023;19(1):165. 37730566 10.1186/s12917-023-03724-y PMC10510151

[88] Ryan EM, et al. Synergistic phage-antibiotic combinations for the control of Escherichia coli biofilms in vitro. FEMS Immunol Med Microbiol. 2012;65(2):395–8. 22524448 10.1111/j.1574-695X.2012.00977.x

[89] Korf IH, et al. In vitro evaluation of a phage cocktail controlling infections with Escherichia coli. Viruses. 2020;12(12):1470. 33352791 10.3390/v12121470 PMC7768485

[90] Costa P, et al. Efficiency of single phage suspensions and phage cocktail in the inactivation of Escherichia coli and Salmonella typhimurium: an in vitro preliminary study. Microorganisms. 2019;7(4):94. 30935094 10.3390/microorganisms7040094 PMC6518180

[91] Battistelli N, et al. In vitro characterization and genome sequencing of two novel lytic phages against Salmonella infantis isolated from poultry feces. Front Microbiol. 2024;15:1479700. 39703709 10.3389/fmicb.2024.1479700 PMC11655500

[92] Shang Y, et al. Isolation and characterization of a novel Salmonella phage vB_SalP_TR2. Front Microbiol. 2021;12:664810. 34234757 10.3389/fmicb.2021.664810 PMC8256156

[93] Pelyuntha W, et al. Effect of novel phage cocktail on Salmonella recovered from broiler sources and its anti-biofilm effect on food contact surface model. Food Control. 2025;169:111000.

[94] Steffan SM, et al. Campylobacter bacteriophage cocktail design based on an advanced selection scheme. Antibiotics. 2022;11(2):228. 35203830 10.3390/antibiotics11020228 PMC8868561

[95] Xiao K, et al. Application of a novel phage vB_CjeM_WX1 to control Campylobacter jejuni in foods. Int J Food Microbiol. 2025;427:110975. 39550792 10.1016/j.ijfoodmicro.2024.110975

[96] Zhang X, et al. A broad host phage, CP6, for combating multidrug-resistant Campylobacter prevalent in poultry meat. Poult Sci. 2024;103(4):103548. 38442560 10.1016/j.psj.2024.103548 PMC10964072

[97] Al-Mohammadi A-R, et al. Isolation and characterization of lytic bacteriophages specific for Campylobacter jejuni and Campylobacter coli. Zoonotic Dis. 2022;2(2):59–72.

[98] Mallick B, Mondal P, Dutta M. Morphological, biological, and genomic characterization of a newly isolated lytic phage Sfk20 infecting Shigella flexneri, Shigella sonnei, and Shigella dysenteriae1. Sci Rep. 2021;11(1):19313. 34588569 10.1038/s41598-021-98910-z PMC8481304

[99] Kim S-H, Adeyemi DE, Park M-K. Characterization of a new and efficient polyvalent phage infecting E. coli O157: H7, Salmonella spp., and Shigella sonnei. Microorganisms. 2021;9(10):2105. 34683426 10.3390/microorganisms9102105 PMC8540833

[100] Choi J, Park S, Chang Y. Development and application of a bacteriophage cocktail for Shigella flexneri biofilm Inhibition on the stainless steel surface. Food Microbiol. 2025;125:104641. 39448151 10.1016/j.fm.2024.104641

[101] Whittle M, et al. A novel bacteriophage with broad host range against clostridioides difficile ribotype 078 supports SlpA as the likely phage receptor. Microbiol Spectr. 2022;10(1):e02295–21. 35107319 10.1128/spectrum.02295-21 PMC8809339

[102] Nale JY, et al. Bacteriophage combinations significantly reduce clostridium difficile growth in vitro and proliferation in vivo. Antimicrob Agents Chemother. 2016;60(2):968–81. 26643348 10.1128/AAC.01774-15 PMC4750681

[103] Meader E, et al. Bacteriophage treatment significantly reduces viable clostridium difficile and prevents toxin production in an in vitro model system. Anaerobe. 2010;16(6):549–54. 20816997 10.1016/j.anaerobe.2010.08.006

[104] Meader E, et al. Evaluation of bacteriophage therapy to control clostridium difficile and toxin production in an in vitro human colon model system. Anaerobe. 2013;22:25–30. 23685029 10.1016/j.anaerobe.2013.05.001

[105] Gwak KM et al. Isolation and characterization of a lytic and highly specific phage against yersinia Enterocolitica as a novel biocontrol agent. 2018. 10.4014/jmb.1808.08001 30270603

[106] Abdel-Haliem ME, Askora A. Isolation and characterization of bacteriophages of Helicobacter pylori isolated from Egypt. Future Virol. 2013;8(8):821–6.

[107] Uchiyama J, et al. Characterization of Helicobacter pylori bacteriophage KHP30. Appl Environ Microbiol. 2013;79(10):3176–84. 23475617 10.1128/AEM.03530-12 PMC3685256

[108] Cuomo P, et al. An innovative approach to control H. pylori-induced persistent inflammation and colonization. Microorganisms. 2020;8(8):1214. 32785064 10.3390/microorganisms8081214 PMC7463796

[109] Nale JY, et al. An optimized bacteriophage cocktail can effectively control Salmonella in vitro and in galleria Mellonella. Front Microbiol. 2021;11:609955. 33552020 10.3389/fmicb.2020.609955 PMC7858669

[110] Youssef RA, et al. Genomic characterization, in vitro, and preclinical evaluation of two microencapsulated lytic phages VB_ST_E15 and VB_ST_SPNIS2 against clinical multidrug-resistant Salmonella serovars. Ann Clin Microbiol Antimicrob. 2024;23(1):17. 38360595 10.1186/s12941-024-00678-3 PMC10870556

[111] Xu J, et al. Molecular characteristics of novel phage vB_ShiP-A7 infecting multidrug-resistant Shigella flexneri and Escherichia coli, and its bactericidal effect in vitro and in vivo. Front Microbiol. 2021;12:698962. 34512574 10.3389/fmicb.2021.698962 PMC8427288

[112] Chaudhary N, et al. Vibrio phage VMJ710 can prevent and treat disease caused by pathogenic MDR V. cholerae O1 in an infant mouse model. Antibiotics. 2023;12(6):1046. 37370365 10.3390/antibiotics12061046 PMC10295236

[113] Xue Y, et al. The yersinia phage X1 administered orally efficiently protects a murine chronic enteritis model against yersinia Enterocolitica infection. Front Microbiol. 2020;11:351. 32210942 10.3389/fmicb.2020.00351 PMC7067902

[114] Vandenheuvel D, Lavigne R, Brüssow H. Bacteriophage therapy: advances in formulation strategies and human clinical trials. Annual Rev Virol. 2015;2(1):599–618. 26958930 10.1146/annurev-virology-100114-054915

[115] Sarker SA, et al. Oral phage therapy of acute bacterial diarrhea with two coliphage preparations: A randomized trial in children from Bangladesh. eBioMedicine. 2016;4:124–37. 26981577 10.1016/j.ebiom.2015.12.023 PMC4776075

[116] Chen WH, et al. Safety and tolerability of shigactive™, a Shigella spp. Targeting bacteriophage preparation, in a phase 1 randomized, Double-Blind, controlled clinical trial. Antibiotics. 2024;13(9):858. 39335031 10.3390/antibiotics13090858 PMC11429168

[117] Ács N, Gambino M, Brøndsted L. Bacteriophage enumeration and detection methods. Front Microbiol. 2020;11:594868. 33193274 10.3389/fmicb.2020.594868 PMC7644846

[118] Koo J, Marshall DL, DePaola A. Antacid increases survival of vibrio vulnificus and vibrio vulnificus phage in a Gastrointestinal model. Appl Environ Microbiol. 2001;67(7):2895–902. 11425699 10.1128/AEM.67.7.2895-2902.2001 PMC92958

[119] Smith HW, Huggins MB, Shaw KM. Factors influencing the survival and multiplication of bacteriophages in calves and in their environment. Microbiology. 1987;133(5):1127–35. 10.1099/00221287-133-5-1127 3309178

[120] Tanji Y, et al. Therapeutic use of phage cocktail for controlling Escherichia coli O157: H7 in Gastrointestinal tract of mice. J Biosci Bioeng. 2005;100(3):280–7. 16243277 10.1263/jbb.100.280

[121] Yin H, et al. Microencapsulated phages show prolonged stability in Gastrointestinal environments and high therapeutic efficiency to treat Escherichia coli O157: H7 infection. Vet Res. 2021;52:1–13. 34521472 10.1186/s13567-021-00991-1 PMC8439058

[122] Clokie MRJ, Kropinski. and R. e.e.h.i.l.g.v.r.e. Lavigne, Bacteriophages Methods and Protocols, Volume 3. 1st ed. 2018;2018.

[123] Ackermann H-W. Bacteriophage electron microscopy. Adv Virus Res. 2012;82:1–32. 22420849 10.1016/B978-0-12-394621-8.00017-0

[124] Brussaard CP, Marie D, Bratbak G. Flow cytometric detection of viruses. J Virol Methods. 2000;85(1–2):175–82. 10716350 10.1016/s0166-0934(99)00167-6

[125] Anderson B, et al. Enumeration of bacteriophage particles: comparative analysis of the traditional plaque assay and real-time QPCR-and nanosight-based assays. Bacteriophage. 2011;1(2):86–93. 22334864 10.4161/bact.1.2.15456 PMC3278645

[126] Gutiérrez D, et al. Typing of bacteriophages by randomly amplified polymorphic DNA (RAPD)-PCR to assess genetic diversity. FEMS Microbiol Lett. 2011;322(1):90–7. 21692832 10.1111/j.1574-6968.2011.02342.x

[127] Morella NM, et al. Rapid quantification of bacteriophages and their bacterial hosts in vitro and in vivo using droplet digital PCR. J Virol Methods. 2018;259:18–24. 29859196 10.1016/j.jviromet.2018.05.007

[128] Wang T, et al. Mass spectrometry enumeration of filamentous M13 bacteriophage. Anal Biochem. 2019;582:113354. 31276652 10.1016/j.ab.2019.113354 PMC6886259

[129] Klumpp J, Fouts DE, Sozhamannan S. Next generation sequencing technologies and the changing landscape of phage genomics. Bacteriophage. 2012;2(3):190–9. 23275870 10.4161/bact.22111 PMC3530529

[130] Federici S, et al. Targeted suppression of human IBD-associated gut microbiota commensals by phage consortia for treatment of intestinal inflammation. Cell. 2022;185(16):2879–e289824. 35931020 10.1016/j.cell.2022.07.003

[131] Colom J, et al. Liposome-encapsulated bacteriophages for enhanced oral phage therapy against Salmonella spp. Appl Environ Microbiol. 2015;81(14):4841–9. 25956778 10.1128/AEM.00812-15 PMC4551199

[132] Dlamini SB, et al. Efficacy of different encapsulation techniques on the viability and stability of diverse phage under simulated gastric conditions. Microorganisms. 2023;11(10):2389. 37894046 10.3390/microorganisms11102389 PMC10608910

[133] Ma Y, et al. Microencapsulation of bacteriophage Felix O1 into chitosan-alginate microspheres for oral delivery. Appl Environ Microbiol. 2008;74(15):4799–805. 18515488 10.1128/AEM.00246-08 PMC2519356

[134] Stanford K, et al. Oral delivery systems for encapsulated bacteriophages targeted at Escherichia coli O157: H7 in feedlot cattle. J Food Prot. 2010;73(7):1304–12. 20615343 10.4315/0362-028x-73.7.1304

[135] Li J, et al. Characterization of a novel siphoviridae Salmonella bacteriophage T156 and its microencapsulation application in food matrix. Food Res Int. 2021;140:110004. 33648237 10.1016/j.foodres.2020.110004

[136] Zhang B, et al. Microencapsulated phage composites with increased Gastrointestinal stability for the oral treatment of Salmonella colonization in chicken. Front Veterinary Sci. 2023;9:1101872. 10.3389/fvets.2022.1101872 PMC9875011 36713855

[137] Dąbrowska K, Górski A, Abedon ST. Bacteriophage pharmacology and immunology. Bacteriophages: Biology, Technology, Therapy, 2021: pp. 295–339.

[138] Abd-Allah IM, et al. Rekindling of a masterful precedent; bacteriophage: reappraisal and future pursuits. Front Cell Infect Microbiol. 2021;11:635597. 34136415 10.3389/fcimb.2021.635597 PMC8201069

[139] Nang SC, et al. Pharmacokinetics/pharmacodynamics of phage therapy: a major hurdle to clinical translation. Clin Microbiol Infect. 2023;29(6):702–9. 36736661 10.1016/j.cmi.2023.01.021

[140] Dąbrowska K, Abedon ST. Pharmacologically aware phage therapy: pharmacodynamic and Pharmacokinetic Obstacles to phage antibacterial action in animal and human bodies. Microbiol Mol Biol Rev. 2019;83(4):00012–19. 10.1128/mmbr 10.1128/MMBR.00012-19 PMC6822990 31666296

[141] Reyneke B, et al. Benefits and challenges of applying bacteriophage biocontrol in the consumer water cycle. Microorganisms. 2024;12(6):1163. 38930545 10.3390/microorganisms12061163 PMC11205630

[142] Bozidis P, et al. Does phage therapy need a pan-phage? Pathogens. 2024;13(6):522. 38921819 10.3390/pathogens13060522 PMC11206709

[143] Kapoor A, et al. Phage therapy: A novel approach against multidrug-resistant pathogens. 3 Biotech. 2024;14(10):256. 39355200 10.1007/s13205-024-04101-8 PMC11442959

[144] Costa P et al. A Game of Resistance: War Between Bacteria and Phages and How Phage Cocktails can be the Solution. Virology, 2024: p. 110209. 10.1016/j.virol.2024.110209 39186863

[145] Chae D. Phage-host-immune system dynamics in bacteriophage therapy: basic principles and mathematical models. Translational Clin Pharmacol. 2023;31(4):167. 10.12793/tcp.2023.31.e17 PMC10772058 38196997

[146] Cervera C. Current landscape on phage therapy in infections: time to leave it behind for good? Clin Microbiol Infect. 2023;29(5):565–7. 36736660 10.1016/j.cmi.2023.01.018

[147] Hussain FA, et al. Rapid evolutionary turnover of mobile genetic elements drives bacterial resistance to phages. Science. 2021;374(6566):488–92. 34672730 10.1126/science.abb1083

[148] Abedon ST, Ecology, Evolutionary Biology of Hindering Phage Therapy. The phage tolerance vs. Phage Resist Bacterial Biofilms Antibiot. 2023;12(2):245. 10.3390/antibiotics12020245 PMC9952518 36830158

[149] Torres-Barceló C, Turner PE, Buckling A. Mitigation of evolved bacterial resistance to phage therapy. Curr Opin Virol. 2022;53:101201. 35180532 10.1016/j.coviro.2022.101201

[150] Seed KD, et al. Evolutionary consequences of intra-patient phage predation on microbial populations. Elife. 2014;3:e03497. 25161196 10.7554/eLife.03497 PMC4141277

[151] Smith HW, Huggins M. Effectiveness of phages in treating experimental Escherichia coli diarrhoea in calves, piglets and lambs. Microbiology. 1983;129(8):2659–75. 10.1099/00221287-129-8-2659 6355391

[152] Sørensen MCH, et al. Phase variable expression of capsular polysaccharide modifications allows Campylobacter jejuni to avoid bacteriophage infection in chickens. Front Cell Infect Microbiol. 2012;2:11. 22919603 10.3389/fcimb.2012.00011 PMC3417653

[153] Chung KM, Liau XL, Tang SS. Bacteriophages and their host range in multidrug-resistant bacterial disease treatment. Pharmaceuticals. 2023;16(10):1467. 37895938 10.3390/ph16101467 PMC10610060

[154] Petrovic Fabijan A, et al. Translating phage therapy into the clinic: recent accomplishments but continuing challenges. PLoS Biol. 2023;21(5):e3002119. 37220114 10.1371/journal.pbio.3002119 PMC10204993

[155] Xu X, Gu P. Overview of phage defense systems in bacteria and their applications. Int J Mol Sci. 2024;25(24):13316. 39769080 10.3390/ijms252413316 PMC11676413

[156] Liu S, et al. Phages against pathogenic bacterial biofilms and Biofilm-Based infections: A review. Pharmaceutics. 2022;14(2):427. 35214158 10.3390/pharmaceutics14020427 PMC8875263

[157] Biggs KRH, et al. Ecological approach to Understanding superinfection Inhibition in bacteriophage. Viruses. 2021;13(7):1389. 34372595 10.3390/v13071389 PMC8310164

[158] Shi K, et al. Structural basis of superinfection exclusion by bacteriophage T4 spackle. Commun Biology. 2020;3(1):691. 10.1038/s42003-020-01412-3 PMC7677548 33214665

[159] Liu Y, et al. Covalent modifications of the bacteriophage genome confer a degree of resistance to bacterial CRISPR systems. J Virol. 2020;94(23):01630–20. 10.1128/jvi 10.1128/JVI.01630-20 PMC7654273 32938767

[160] Smirnov S et al. Fluorescence microscopy study of the effect of Esp1396I restriction-modification system proteins concentrations on protection against lambda phage. in Journal of Physics: Conference Series. 2018. IOP Publishing.

[161] Borges AL, et al. Bacterial alginate regulators and phage homologs repress CRISPR–Cas immunity. Nat Microbiol. 2020;5(5):679–87. 32203410 10.1038/s41564-020-0691-3 PMC7190418

[162] Lopatina A, Tal N, Sorek R. Abortive infection: bacterial suicide as an antiviral immune strategy. Annual Rev Virol. 2020;7(1):371–84. 32559405 10.1146/annurev-virology-011620-040628

[163] Feyereisen M et al. Identification of a prophage-encoded abortive infection system in Levilactobacillus brevis. 2020.

[164] Montso PK, Mlambo V, Ateba CN. Efficacy of novel phages for control of multi-drug resistant Escherichia coli O177 on artificially contaminated beef and their potential to disrupt biofilm formation. Food Microbiol. 2021;94:103647. 33279072 10.1016/j.fm.2020.103647

[165] Malik S, Nehra K, Rana J. Bacteriophage cocktail and phage antibiotic synergism as promising alternatives to conventional antibiotics for the control of multi-drug-resistant uropathogenic Escherichia coli. Virus Res. 2021;302:198496. 34182014 10.1016/j.virusres.2021.198496

[166] Maszewska A, et al. Use of polyvalent bacteriophages to combat biofilm of proteus mirabilis causing catheter-associated urinary tract infections. J Appl Microbiol. 2018;125(5):1253–65. 29924909 10.1111/jam.14026

[167] Townsend EM, Moat J, Jameson E. CAUTI’s next top model–Model dependent Klebsiella biofilm Inhibition by bacteriophages and antimicrobials. Biofilm. 2020;2:100038. 33381752 10.1016/j.bioflm.2020.100038 PMC7762788

[168] Chaudhry WN, et al. Synergy and order effects of antibiotics and phages in killing Pseudomonas aeruginosa biofilms. PLoS ONE. 2017;12(1):e0168615. 28076361 10.1371/journal.pone.0168615 PMC5226664

[169] Kumaran D, et al. Does treatment order matter? Investigating the ability of bacteriophage to augment antibiotic activity against Staphylococcus aureus biofilms. Front Microbiol. 2018;9:127. 29459853 10.3389/fmicb.2018.00127 PMC5807357

[170] Huss P, Raman S. Engineered bacteriophages as programmable biocontrol agents. Curr Opin Biotechnol. 2020;61:116–21. 31862543 10.1016/j.copbio.2019.11.013 PMC7103757

[171] Li M, et al. Recombination of T4-like phages and its activity against pathogenic Escherichia coli in planktonic and biofilm forms. Virol Sin. 2020;35:651–61. 32451882 10.1007/s12250-020-00233-2 PMC7736419

[172] Lu TK, Collins JJ. Dispersing biofilms with engineered enzymatic bacteriophage. Proc Natl Acad Sci. 2007;104(27):11197–202. 17592147 10.1073/pnas.0704624104 PMC1899193

[173] Born Y, et al. Engineering of bacteriophages Y2:: dpoL1-C and Y2:: LuxAB for efficient control and rapid detection of the fire blight pathogen, erwinia Amylovora. Appl Environ Microbiol. 2017;83(12):e00341–17. 28389547 10.1128/AEM.00341-17 PMC5452800

[174] Azeredo J, Garcia P, Drulis-Kawa Z. Targeting biofilms using phages and their enzymes. Curr Opin Biotechnol. 2021;68:251–61. 33714050 10.1016/j.copbio.2021.02.002

[175] Vázquez R, García P. Synergy between two chimeric lysins to kill Streptococcus pneumoniae. Front Microbiol. 2019;10:1251. 31231338 10.3389/fmicb.2019.01251 PMC6560164

[176] Letrado P, et al. Bactericidal synergism between antibiotics and phage endolysin Cpl-711 to kill multidrug-resistant Pneumococcus. Future Microbiol. 2018;13(11):1215–23. 30238774 10.2217/fmb-2018-0077 PMC6190277

[177] Stachler E, Kull A, Julian TR. Bacteriophage treatment before chemical disinfection can enhance removal of plastic-surface-associated Pseudomonas aeruginosa. Appl Environ Microbiol. 2021;87(20):e00980–21. 34347517 10.1128/AEM.00980-21 PMC8478462

[178] Bone S, et al. Physisorption and chemisorption of T4 bacteriophages on amino functionalized silica particles. J Colloid Interface Sci. 2018;532:68–76. 30077067 10.1016/j.jcis.2018.07.107

[179] Cook BW, Hynes AP. Re-evaluating what makes a phage unsuitable for therapy. Npj Antimicrobials Resist. 2025;3(1):1–5. 10.1038/s44259-025-00117-z PMC12122733 40442282

[180] Borysowski J, Ehni HJ, Górski A. Ethics codes and use of new and innovative drugs. Br J Clin Pharmacol. 2019;85(3):501–7. 30536603 10.1111/bcp.13833 PMC6379208

[181] Fauconnier A. Phage therapy regulation: from night to dawn. Viruses. 2019;11(4):352. 30999559 10.3390/v11040352 PMC6521264

[182] Żaczek M, et al. Phage therapy in Poland–a centennial journey to the first ethically approved treatment facility in Europe. Front Microbiol. 2020;11:1056. 32582061 10.3389/fmicb.2020.01056 PMC7291835

[183] Knezevic P, et al. Advances in phage therapy: present challenges and future perspectives. Front Microbiol. 2021;12:701898. 34220788 10.3389/fmicb.2021.701898 PMC8248810

[184] Lin RC, et al. Phage biobank: present challenges and future perspectives. Curr Opin Biotechnol. 2021;68:221–30. 33581425 10.1016/j.copbio.2020.12.018

[185] Suh GA, et al. Considerations for the use of phage therapy in clinical practice. Antimicrob Agents Chemother. 2022;66(3):e02071–21. 35041506 10.1128/aac.02071-21 PMC8923208

[186] Sulakvelidze A, Alavidze Z, Morris JG Jr. Bacteriophage therapy. Antimicrob Agents Chemother. 2001;45(3):649–59. 11181338 10.1128/AAC.45.3.649-659.2001 PMC90351

[187] Międzybrodzki R, et al. Clinical aspects of phage therapy. Adv Virus Res. 2012;83:73–121. 22748809 10.1016/B978-0-12-394438-2.00003-7

[188] Górski A, et al. Phage therapy: what have we learned? Viruses. 2018;10(6):288. 29843391 10.3390/v10060288 PMC6024844

[189] Górski A, et al. Phage therapy: current status and perspectives. Med Res Rev. 2020;40(1):459–63. 31062882 10.1002/med.21593

[190] Pirnay J-P, et al. The phage therapy paradigm: prêt-à-porter or sur-mesure? Pharm Res. 2011;28:934–7. 21063753 10.1007/s11095-010-0313-5

[191] Pirnay J-P, et al. The magistral phage. Viruses. 2018;10(2):64. 29415431 10.3390/v10020064 PMC5850371

[192] Aslam S et al. Lessons learned from the first 10 consecutive cases of intravenous bacteriophage therapy to treat Multidrug-Resistant bacterial infections at a single center in the united States. Open Forum Infect Dis, 2020;7(9). 10.1093/ofid/ofaa389 PMC7519779 33005701

[193] Aziza F. Comparison review of two regulatory agencies regulation: therapeutic goods administration (TGA) and the European medicine agency (EMA) in relation to good manufacturing practice (GMP) guideline. Majalah Farmaseutik. 2021;17(2):243–8.

[194] (MHRA). M.a.H.P.R.A., Regulatory considerations for therapeutic use of bacteriophages in the UK. Available online: https://www.gov.uk/government/publications/regulatory-considerations-for-therapeutic-use-of-bacteriophages-in-the-uk published on 25 June 2025.

[195] Zalewska-Piątek B, Therapy—Challenges P. Opportunities and future prospects. Pharmaceuticals. 2023;16(12):1638. 38139765 10.3390/ph16121638 PMC10747886

[196] Luong T, et al. Standardized bacteriophage purification for personalized phage therapy. Nat Protoc. 2020;15(9):2867–90. 32709990 10.1038/s41596-020-0346-0

[197] Branston SD, Wright J, Keshavarz-Moore E. A non‐chromatographic method for the removal of endotoxins from bacteriophages. Biotechnol Bioeng. 2015;112(8):1714–9. 25728530 10.1002/bit.25571

[198] Hodyra-Stefaniak K, et al. Mammalian Host-Versus-Phage immune response determines phage fate in vivo. Sci Rep. 2015;5(1):14802. 26440922 10.1038/srep14802 PMC4594097

[199] Sunderland KS, Yang M, Mao C. Phage-enabled nanomedicine: from probes to therapeutics in precision medicine. Angew Chem Int Ed. 2017;56(8):1964–92. 10.1002/anie.201606181 PMC5311110 27491926

[200] Kim B-o, et al. Phage-derived antibacterials: Harnessing the simplicity, plasticity, and diversity of phages. Viruses. 2019;11(3):268. 30889807 10.3390/v11030268 PMC6466130

[201] Skurnik M, Pajunen M, Kiljunen S. Biotechnological challenges of phage therapy. Biotechnol Lett. 2007;29:995–1003. 17364214 10.1007/s10529-007-9346-1

[202] Rehman S, et al. The dawn of phage therapy. Rev Med Virol. 2019;29(4):e2041. 31050070 10.1002/rmv.2041

[203] Kuang X, et al. Applications of bacteriophages in precision engineering of the human gut Microbiome. Eng Microbiol. 2025;5(1):100189. 40538713 10.1016/j.engmic.2025.100189 PMC12173823

[204] Loc-Carrillo C, Abedon ST. Pros and cons of phage therapy. Bacteriophage. 2011;1(2):111–4. 22334867 10.4161/bact.1.2.14590 PMC3278648

[205] Meng L, et al. Nanocapping-enabled charge reversal generates cell-enterable endosomal-escapable bacteriophages for intracellular pathogen Inhibition. Sci Adv. 2022;8(28):peabq2005. 10.1126/sciadv.abq2005 PMC11581130 35857522

[206] Bichet MC, et al. Mammalian cells internalize bacteriophages and use them as a resource to enhance cellular growth and survival. PLoS Biol. 2023;21(10):e3002341. 37883333 10.1371/journal.pbio.3002341 PMC10602308

[207] Wang H, et al. Phage-based delivery systems: engineering, applications, and challenges in nanomedicines. J Nanobiotechnol. 2024;22(1):365. 10.1186/s12951-024-02576-4 PMC11197292 38918839

[208] Gondil VS, Chhibber S. Bacteriophage and endolysin encapsulation systems: a promising strategy to improve therapeutic outcomes. Front Pharmacol. 2021;12:675440. 34025436 10.3389/fphar.2021.675440 PMC8138158

[209] Pinto AM, et al. The clinical path to deliver encapsulated phages and lysins. FEMS Microbiol Rev. 2021;45(5):fuab019. 33784387 10.1093/femsre/fuab019

[210] Xu Y. Phage and phage lysins: new era of bio-preservatives and food safety agents. J Food Sci. 2021;86(8):3349–73. 34302296 10.1111/1750-3841.15843

